# Stepwise Crystalline Structural Transformation in 0D Hybrid Antimony Halides with Triplet Turn-on and Color-Adjustable Luminescence Switching

**DOI:** 10.34133/research.0094

**Published:** 2023-03-30

**Authors:** Jian-Qiang Zhao, Yue-Yu Ma, Xue-Jie Zhao, Yu-Jia Gao, Zi-Yan Xu, Pan-Chao Xiao, Cheng-Yang Yue, Xiao-Wu Lei

**Affiliations:** School of Chemistry, Chemical Engineer and Materials, Jining University, Qufu, Shandong 273155, P. R. China.

## Abstract

Intelligent stimuli-responsive fluorescence materials are extremely pivotal for fabricating luminescent turn-on switching in solid-state photonic integration technology, but it remains a challenging objective for typical 3-dimensional (3D) perovskite nanocrystals. Herein, by fine-tuning the accumulation modes of metal halide components to dynamically control the carrier characteristics, a novel triple-mode photoluminescence (PL) switching was realized in 0D metal halide through stepwise single-crystal to single-crystal (SC-SC) transformation. Specifically, a family of 0D hybrid antimony halides was designed to exhibit three distinct types of PL performance including nonluminescent [Ph_3_EtP]_2_Sb_2_Cl_8_ (**1**), yellow-emissive [Ph_3_EtP]_2_SbCl_5_·EtOH (**2**), and red-emissive [Ph_3_EtP]_2_SbCl_5_ (**3**). Upon stimulus of ethanol, **1** was successfully converted to **2** through SC-SC transformation with enhanced PL quantum yield from ~0% to 91.50% acting as “turn-on” luminescent switching. Meanwhile, reversible SC-SC and luminescence transformation between **2** and **3** can be also achieved in the ethanol impregnation–heating process as luminescence vapochromism switching. As a consequence, a new triple-model turn-on and color-adjustable luminescent switching of off–on^I^–on^II^ was realized in 0D hybrid halides. Simultaneously, wide advanced applications were also achieved in anti-counterfeiting, information security, and optical logic gates. This novel photon engineering strategy is expected to deepen the understanding of dynamic PL switching mechanism and guide development of new smart luminescence materials in cutting-edge optical switchable device.

## Introduction

As molecule-level artificial intelligent photoelectric switches, smart stimulus-responsive luminescence materials have attracted intensive attention in the coming photonic era for both science and technology [1–5]. Generally, smart luminescence materials are capable of displaying adjustable or switchable light-emitting characteristics of wavelength, intensity, and lifetime upon external chemical or physical stimuli including force, light, small gas or molecular, metal ions, organic vapor, pH, temperature, and X-ray radiation [6–10]. By managing these external stimuli as inputs, switchable fluorescence can be precisely controlled as responsive output to realize dynamic optical signal with wide applications in cutting-edge photonic devices, such as sensing, optical memory, data storage, logic gate, and anti-counterfeiting [11–15].

In the past several years, inorganic 3D perovskite nanocrystals (PNCs) of CsPbX_3_ (X = Cl, Br, I) have emerged as one of the most promising photoluminescence (PL) materials with multiple unique superiorities including tunable bandgaps and emission wavelength covering the entire visible spectral range, high color purity, and PL quantum yield (PLQY) at room temperature [16–18]. More importantly, the luminescence of 3D PNCs is highly sensitive to various external stimuli with diversified PL switching behaviors including mechanochromism, thermochromism, vapochromism, photochromism, and hydrochromism [19–21]. Despite the sensitive switchable PL performance, 3D PNC-based fluorescence switches remain to unavoidably confront with some fatal shortcomings. On the one hand, most of the 3D PNC luminescence switches play the functions only in solution phase but fail at the solid state due to notorious aggregation-caused quenching (ACQ) effect of colloidal nanoparticles [22,23]. In fact, the solid-state PL switch is more suitable for practical applications but is rarely reported until now. On the other hand, 3D PNC is prone to decompose or convert into nonluminescent phase (such as CsPb_2_X_5_) once exposed to moisture, oxygen, and heat due to the weak ionic bond nature and lower formation energy [24,25]. As a result, the fluorescence of 3D PNCs is easily quenched by these external stimuli with serious instability, which only represents as turn-off mode fluorescence switch. Relatively speaking, rare turn-on or reversible on-off mode PL switch is realized, but it is more applicable for high-resolution optical imaging. Therefore, although 3D PNCs can be utilized as fluorescence sensors due to impressible PL performance, it remains an extremely challenging objective to realize solid-state fluorescence turn-on switch in perovskite chemistry, let alone the multiple-mode switchable luminescence.

Comparing with typical 3D PNCs, a newly emerged candidate of organic–inorganic hybrid low-dimensional halide perovskite (LDHP) not only possesses ultrahigh structural stability and luminescence efficiency but also combines multiple advantages of diversified luminescent performance and stimuli-responsive fluorescence switch [26,27]. Benefiting from the spatial separation and quantum confinement effects of organic matrix toward discrete metal halide species, single-crystalline LDHPs fortunately escape from the damage of ACQ with ultrahigh photophysical stability and luminescence efficiency at solid state [28,29]. As a consequence, even without any external protection management, LDHPs remain to intrinsically prevent the aggregation of nanoscale luminescent centers, resulting in higher luminescent stabilities than colloid 3D PNCs. Meanwhile, the synergistic effect of versatile organic and inorganic components delivers diversified structures of LDHPs, which provides more opportunities to modulate the PL properties. Different from the exclusive bandgap determined narrow light emission of 3D PNC, LDHP generally displays more adjustable self-trapped exciton (STE)-induced broadband emission due to electron–phonon coupling in deformable crystal lattice [30–33]. Even through finely regulating the structural assembly characteristics of halide anions with designated components, the luminescence properties of LDHPs still can be modulated on a large scale. For example, previous studies have demonstrated that higher emission efficiency is strongly associated with lower dimensionality and concentration of anionic species [34,35]. Specifically, lower dimensionality causes stronger quantum confinement effects and highly localized excitons, and lower concentration of anionic centers weakens the energy interaction between them, which would result in higher exciton binding energy and higher luminescence efficiency [29]. Hence, it is possible to ignite the luminescence of LDHP by separating the metal halide anions over critical value as turn-on mode PL switch. Besides, the Stokes shift of emission property is positively related to the structural distortion level of metal halide anions, which provides feasibility to modulate the emission position by controlling the structural deformability as color-tunable PL switch [36–39]. Finally, the flexible and soft crystal structure of LDHP also exhibits facile single-crystal to single-crystal (SC-SC) transformation upon various external stimuli accompanied by diversified PL switches, such as thermally induced transition between [Bzmim]_3_SbCl_6_ (green, Bzmim = 1-benzyl-3-methylimidazolium) and [Bzmim]_2_SbCl_5_ (red), and [PP14]_2_[PbBr_4_] (blue, PP14 = N-butyl-N-methylpiperidinium) and [PP14]_9_[PbBr_4_]_2_[Pb_3_Br_11_] (green) [40,41]. Therefore, combined superiorities of highly adjustable multiple-component structure, structurally sensitive fluorescence response, and facile SC-SC transformation upon external stimuli imply a possibility of realizing more smart fluorescence switch by virtue of the stimuli-responsive structural reassembly of LDHP.

Despite high expectation, the vast majority of smart luminescent LDHPs overwhelmingly dependent on the slight shift of emission wavelength with low contrast acting as color-tunable PL switch, few approach has shown to realize PL off–on switch [42–44]. The research deficiency attracts intensive attention to further explore more smart fluorescence switch by finely tuning the local structure of LDHP. Based on these considerations, we propose to utilize external stimuli-responsive SC-SC transformation as structural reassembly strategy to dynamically modulate the PL properties of LDHP aiming to realize the target of “small input yields big output” at the molecular level. To demonstrate the feasibility of this design strategy, we select 0D hybrid antimony halide as structural prototype considering the unique advantages of abundant coordination configurations of [SbX_5_]^2−^ and [SbX_6_]^3−^, diversified structural architecture, higher PLQYs, oxidation resistance abilities, nontoxicity, impressible soft crystal structure, and smart PL switching. Herein, by using the same ethyltriphenylphosphonium ([Ph_3_EtP]^+^) as bulk cation, we successfully realized three distinct PL performances in a family of hybrid antimony halide platform including nonluminescent [Ph_3_EtP]_2_Sb_2_Cl_8_, yellow-emissive [Ph_3_EtP]_2_SbCl_5_·EtOH, and red-emissive [Ph_3_EtP]_2_SbCl_5_. Along with the successive ethanol (EtOH) impregnation–drying process, stepwise SC-SC transformations were consecutively achieved with important structural reassembly from condensed [Sb_4_Cl_16_]^4−^ tetramer to discrete [SbCl_5_]^2−^ unit. This structural evolution not only inhibits the concentration quenching effect but also improves the structural distortion level of anionic species, accompanied by twice PL transformation from nonluminescence to yellow and then red light emission as triplet turn-on and color-tunable PL switching. To the best of our knowledge, this work represents a rare triple-mode off–on^I^–on^II^ PL switch in 0D hybrid halides with advanced applications in anti-counterfeiting, information encryption–decryption, and optical logic gate.

## Results

### Single-crystal structure analysis and PL properties

**Fig. 1.**
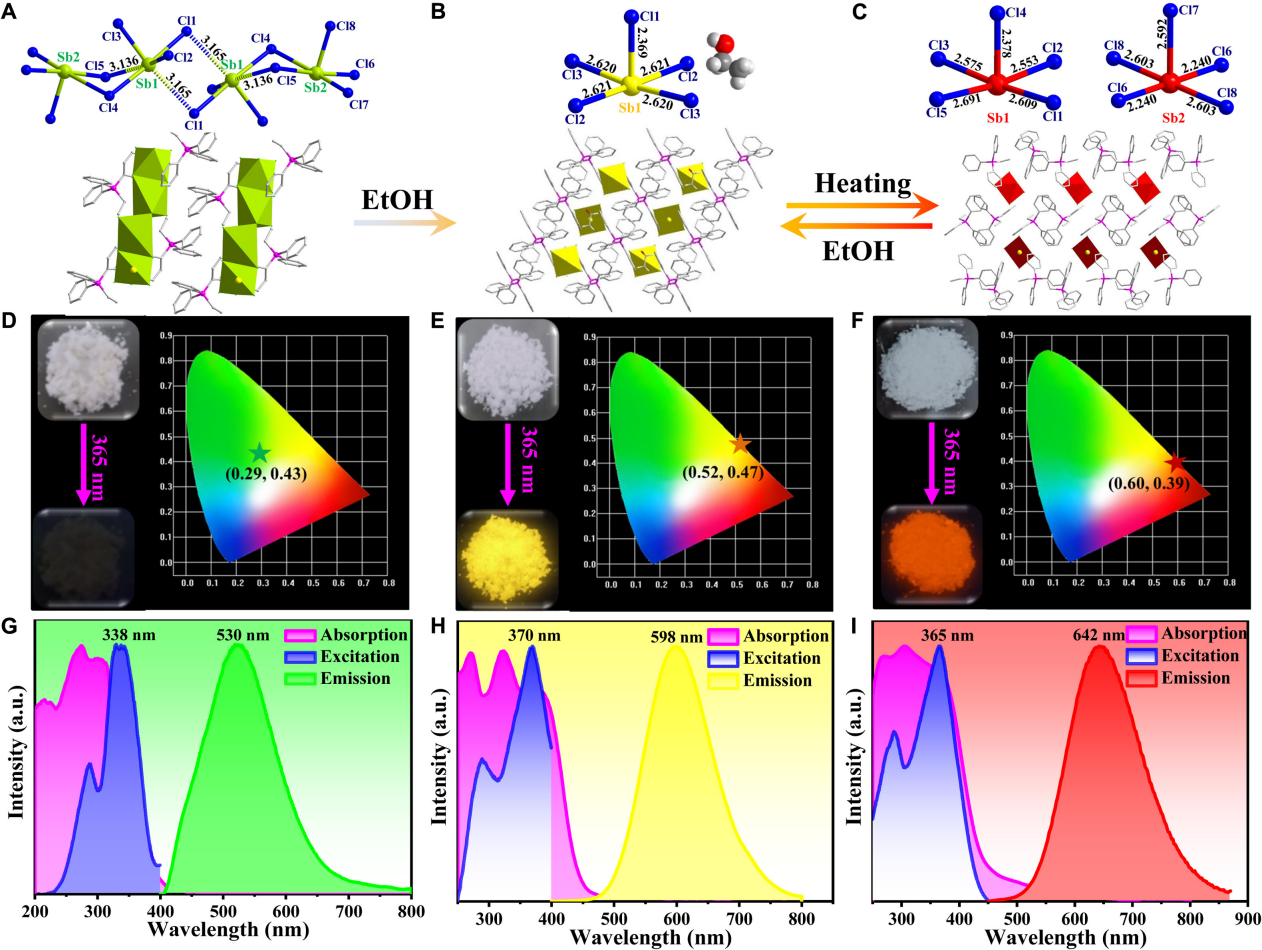
Crystal structures of compounds 1 (A), 2 (B), and 3 (C); photo-images of single crystals under sunlight and 365 nm UV light, as well as Commission Internationale de l’Eclairage (CIE) chromaticity coordinates for compounds 1 (D), 2 (E), and 3 (F); solid-state UV–Vis absorption; and PLE and PL spectra of compounds 1 (G), 2 (H), and 3 (I) at 300 K.

Compounds **1** and **2** were prepared through typical wet-chemistry solution methods with the same precursor materials in different mixed solvents. Based on single-crystal X-ray diffraction, crystal structures of **1** and **2** crystallize in the monoclinic space groups of *P*2_1_/n and *C*2/c containing [Sb_4_Cl_16_]^4−^ tetramer and [SbCl_5_]^2−^ square pyramid as basic building units, respectively. As shown in Fig. 1A, the asymmetric unit of **1** contains two crystallographically independent Sb atoms, both of which are coordinated by five Cl atoms in square pyramidal coordination geometries with Sb–Cl bond distances in the large range of 2.3818(5) to 2.9693(5) Å. [Sb(1)Cl_5_] and [Sb(2)Cl_5_] units are condensed via edge-sharing to form [Sb_2_Cl_8_] dimer, which is further interconnected via weak Sb–Cl bonds (3.165 Å) into [Sb_4_Cl_16_]^4−^ tetramer charge balanced by [Ph_3_EtP]^+^ cation. The crystal structure of compound **2** contains discrete [SbCl_5_]^2−^ square pyramids with normal Sb–Cl bond distances of 2.3688(4) to 2.6209(3) Å, which are spatially separated by [Ph_3_EtP]^+^ cation with guest EtOH locating in the free space (Fig. 1B). Differently, single crystal of compound **3** was obtained from the SC-SC transformation of **2** via solid-state recrystallization reaction. Along with the lost of EtOH molecule, the structural reassembly leads to another type of packing arrangement of [SbCl_5_]^2−^ and [Ph_3_EtP]^+^ blocks in triclinic space group (*P*-1) accompanied by variant Sb–Cl bond distances (2.240-2.691 Å) (Fig. 1C). Overall, all the compounds **1** to **3** belong to typical 0D hybrid structures with discrete [Sb_4_Cl_16_]^4−^ or [SbCl_5_]^2−^ as luminescent center periodically embedding in the organic [Ph_3_EtP]^+^ matrices. As a consequence, different structural assembly characteristics including geometry configuration, distortion degree, and stacking manner of anionic species synergistically induce the distinct PL performance of these 0D hybrid halides as following detailed discussion (Figs. S1 to S3). The sample purity of compounds **1** to **3** was validated by coincident powder X-ray diffraction (PXRD) patterns and simulated data from single-crystal X-ray diffractions (Fig. S4). The elemental mapping from scanning electron microscopy (SEM) demonstrates the presence and uniform distribution of Sb, Cl, C, and P elements on the surface of block single crystals for all compounds (Figs. S5, S15, and S16).

**Table.** Summary of photophysical properties of [Ph_3_EtP]_2_Sb_2_Cl_8_ (1), [Ph_3_EtP]_2_SbCl_5_·EtOH (2), and [Ph_3_EtP]_2_SbCl_5_ (3) ^a^.

	*λ*_ex_ [nm]	*λ*_em_ [nm]	CIE	Stokes shift [nm]	FWHM [nm]	*Φ* (%)	*τ*_av_ (μs)
[Ph_3_EtP]_2_Sb_2_Cl_8_	338	530	(0.29, 0.43)	182	136	~0	3.450
[Ph_3_EtP]SbCl_5_·EtOH	370	598	(0.52, 0.47)	228	123	91.5	3.527
[Ph_3_EtP]SbCl_5_	365	642	(0.60, 0.39)	277	146	64.47	4.364

^a^*λ*_ex_ and *λ*_em_ represent the maximum excitation and emission wavelengths, respectively; *Φ* is the PLQY; and *τ*_av_ is the PL lifetime.

**Fig. 2.**
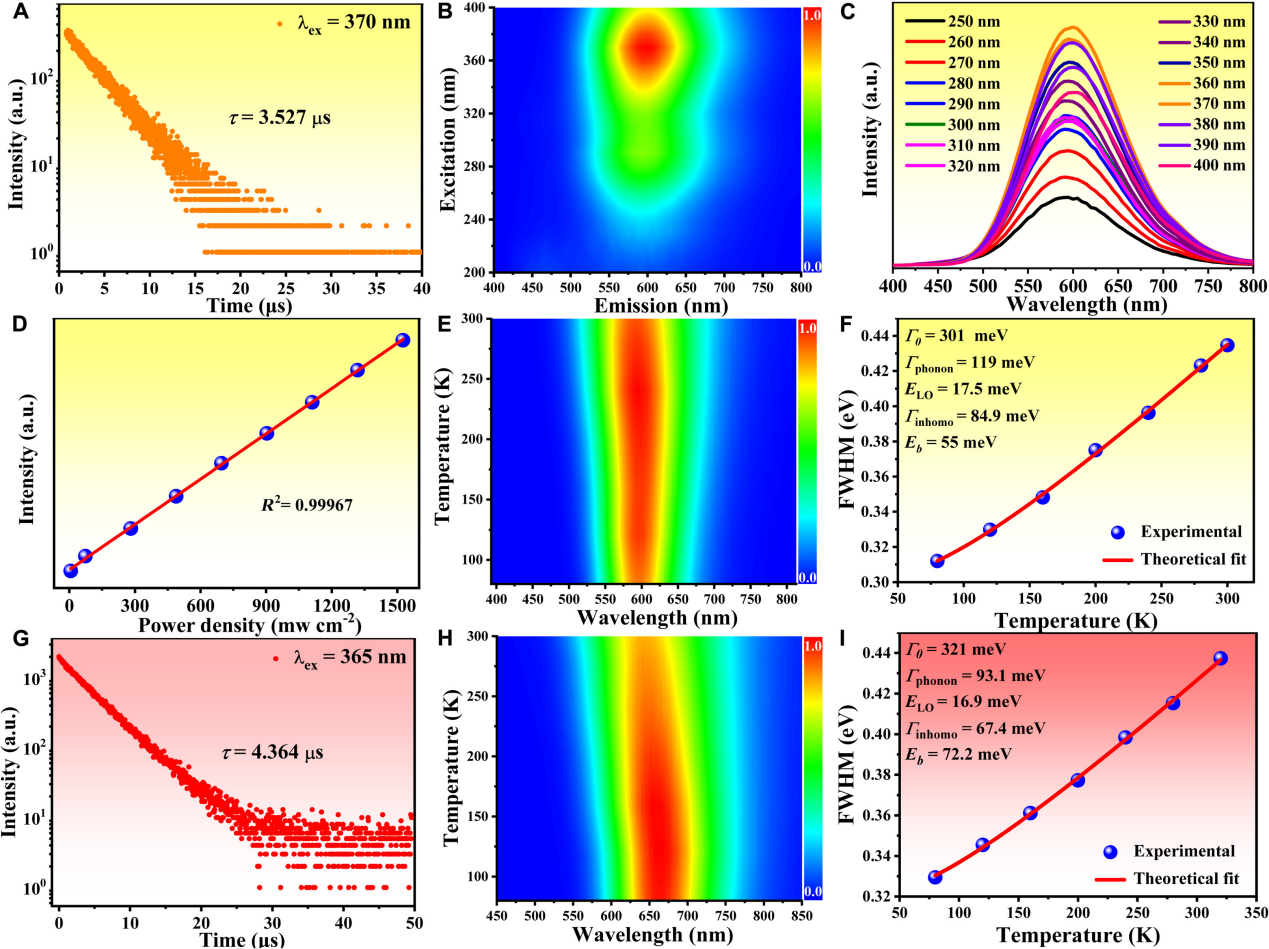
PL property characterizations of compounds 2 and 3: PL decay curve of 2 (A), 3D PL consecutive map of 2 (B), excitation wavelength-dependent PL spectra of 2 (C), excitation power-dependent PL emission intensity of 2 (D), temperature-dependent PL emission spectra of 2 (E), experimental and fitted temperature-dependent FWHM of 2 (F), PL decay curve of 3 (G), temperature-dependent PL emission spectra of 3 (H), and experimental and fitted temperature-dependent FWHM of 3 (I).

All the transparent bulk crystals of compounds **1**, **2**, and **3** are colorless under ambient sunlight, but **1** is completely nonluminescent, while **2** and **3** exhibit strong yellow and red light emissions under irradiation of 365 nm ultraviolet (UV) light as shown in Fig. 1D to F. To obtain the specific comprehension, the optical properties of compounds **1** to **3** are characterized systematically based on solid-state absorption, excitation, and emission spectra. As shown in Fig. 1G to I and Fig. S6, the UV–visible (UV–Vis) absorption spectra of compounds **1** to **3** show multiple absorbance bands, which can be attributed to the electron conversion between the *sp* excited state and *s*^2^ ground state, including the low-energy spin-forbidden transition of ^1^*S*_0_-^3^*P*_1_, the intermediate-energy spin-forbidden transition of ^1^*S*_0_-^3^*P*_2_, and the high-energy spin-allowed transition of ^1^*S*_0_-^1^*P*_1_ [45]. The absorption features for **1** to **3** are similar to those of (C_6_H_5_CH_2_NH_3_)_3_SbBr_6_ and [(C_6_H_22_N_4_)_2_(Sb_2_Cl_10_)(SbCl_6_)(Cl)_2_(H_3_O)]·(3H_2_O) [46,47]. Best fitting based on Tauc function gives direct bandgaps of 3.26, 2.95, and 2.82 eV for **1** to **3**, respectively (Fig. S6). All the PL excitation (PLE) spectra of compounds **1** to **3** feature two dominant bands corresponding to electronic transition of ^1^*S*_0_ → ^1^*P*_1_ (below 300 nm) and ^1^*S*_0_ → ^3^*P*_1_ (above 300 nm), respectively, which are in accord with the UV−vis absorption spectra [48]. Subsequently, the strongest excitation wavelength is selected as light source to investigate the PL emission properties. Despite the invisible luminescence of compound **1** by the naked eye, spectroscopy analysis gives a very weak broadband green light emission at about 530 nm (CIE chromaticity coordinates: 0.29, 0.43) with negligible PLQY of ~0% (Fig. 1G). Based on previously reported work, the weak luminescence of **1** is mainly related to the higher nonradiative transition rate due to strong concentration-caused quenching effect in [Sb_4_Cl_16_]^4−^ tetramer [29,49–52]. Specifically, the shorter Sb···Sb distance (4.021 and 4.148 Å) within [Sb_4_Cl_16_]^4−^ tetramer enhances the electronic coupling and nonradiative energy transfer between adjacent [SbCl_5_] center, and therefore, larger concentration of optically active centers quenches the radiative recombination. Distinctly, compound **2** displays a perfect Gauss-shaped broadband yellow light emission centered at 598 nm with Stokes shift of 228 nm, full width at half maximum (FWHM) of 123 nm, and CIE chromaticity coordinates of (0.52, 0.47) (Fig. 1H). Comparing with the negligible PLQY of compound **1**, the strong yellow emission of compound **2** is verified by near-unity PLQY of 91.5%, which represents a high value in all yellow light-emitting perovskites (Fig. S8 and Table S1). To reveal the dynamic photophysical process, the time-resolved PL spectrum monitoring the maximum emission wavelength is performed at 300 K. The PL decay curve of compound **1** can be fitted by a double-exponential function giving an average lifetime of 3.45 μs (Fig. S7). For compound **2**, the PL decay curve delivers single exponential function fitting with lifetime of 3.527 μs (Fig. 2A). Relative to yellow-emissive compound **2**, the PL spectrum of compound **3** displays a significant red-shift with maximum emission wavelength of 642 nm, Stokes shift of 277 nm, FWHM of 146 nm, and CIE chromaticity coordinates of (0.60, 0.39) corresponding to red light (Fig. 1I). The larger Stokes shift and wider FWHM of **3** than **2** can be interpreted by the higher distortion level of anionic [SbCl_5_]^2−^ units as illustrated in Table S6, and such phenomenon has been confirmed in many 0D hybrid halide perovskites [53]. The strong red light emission of compound **3** is proved by promising PLQY of 64.47%, which is comparable with the vast majority of red light-emitting 0D hybrid metal halides [53–55]. Based on time-resolved spectrum, the PL decay curve is fitted by a single exponential function giving a lifetime of 4.364 μs at 300 K, which is slightly longer than that of compound **2** due to the larger distortion level of [SbCl_5_]^2−^ unit (Fig. 2G).

### PL mechanism

In order to confirm the PL nature of these broadband light emissions, varied wavelength-dependent spectroscopy was performed for compounds **2** and **3** as examples due to the highly efficient emitting performance. Apparently, there is no obvious difference in the PLE and PL spectra under varied emission and excitation wavelength for compound **2**, respectively, corresponding to one dominant emitting center in 3D PL map, which suggests the yellow light emission originates from the relaxation of single excited state (Fig. 2B and C and Fig. S9). Compound **3** also delivers the same dominant red light emission in the whole UV light spectral range accompanied by one additional weak higher-energy emission at about 500 nm under the excitation of higher-energy UV light (<350 nm) (Figs. S10 to S12). We further compare the PL emission spectra of bulk crystals and microscale powders for both compounds considering the fact that the surface defect-associated broadband light emission shows a strong dependence on particle size and is readily quenched by particle aggregation (Figs. S13 and S14). The identical PL emission spectral profiles with slightly decreased intensities of microscale powders exclude the possibility of surface defect-generated broadband light emissions (Figs. S15 and S16) [56]. Additionally, the PL emission intensities of bulk crystals exhibit linear dependence on the excitation power, which rule out the emissions from permanent defects (Fig. 2D and Fig. S17) [57]. To further shed light on the underlying mechanism of broadband light emission, temperature-dependent PL emission spectra were carried out from 80 to 300 K (Fig. 2E and H). In the cooling process, there is not any splitting behavior in the emission spectra, further indicating that the broadband light emissions should originate from the single excited state. Along with the temperature decreasing, the emission intensity of compound **2** initially increases from 300 to 240 K and then slowly decreases until 80 K (Fig. S18). The decreased trend of emission intensity from 240 to 80 K is termed as anti-thermal quenching, which is related to the participation of shallow trapped states arising from the structural defects based on the previously reported work, such as [DMPZ]_2_SbCl_7_∙2H_2_O (DMPZ = N,N′-dimethylpiperazine) and (C_9_NH_20_)_2_SnBr_4_, Mn@CsPbCl_3_ [49,58,59]. For compound **3**, the dominant emission wavelength exhibits a slight red-shift monotonically from 642 to 664 nm with temperature decreasing, which can be attributed to the electron–phonon interaction (Fig. S19). That is, the electron–phonon interaction weakens as the temperature decreases, resulting in a reduction of bandgap and red-shift of emission wavelength. At the same time, the emission intensity gradually increases due to the inhibited nonradiative recombination. Based on the theoretical fitting of temperature-dependent emission intensity according to famous Arrhenius-type equation, the exciton activation energy (*E*_a_) is calculated to be 52.93 meV, which is much larger than the room-temperature thermal ionization energy (~26 meV) [60]. Higher *E*_a_ illustrates that excited electrons difficultly overcome the energy barrier of nonradiative transitions, thus generating stable STEs and strong luminescence at room temperature. Notably, both the PL emission spectra of compounds **2** and **3** broaden gradually with temperature increasing maybe due to the enhanced electron–phonon coupling effect in soft crystal lattice. To quantify the contribution of electron–phonon coupling, the carrier–phonon coupling constant (*Γ*_phonon_) and the corresponding phonon energy (*E*_LO_) can be estimated by theoretically fitting the temperature-dependent FWHM according to the longitudinal optical phonon broadening model:

$$\Gamma \left(T\right)=\Gamma_{0}+\Gamma_{\text{phonon}}\;{\left(e^{\frac{E_{\mathrm{LO}}}{k_{B}T}}-1\right)}^{-1}+\Gamma_{\text{inhomo}}\;e^{-\frac{E_{b}}{k_{B}T}}$$

where *Γ*_0_ is the FWHM of 0 K and *E*_LO_ and *E*_b_ represent the energy of the longitudinal–optical phonon and average binding energy of the trap states, respectively. *Γ*_phonon_ and *Γ*_inhomo_ represent the relative contributions of electron–phonon coupling and inhomogeneous broadening induced by trapped states, respectively [61,62]. The best fittings give *Γ*_phonon_ of 119 and 93.1 meV for compounds **2** and **3**, respectively, which are comparable with most recently reported 0D halides demonstrating predominant contribution of electron–phonon interaction for the PL emission broadening (Fig. 2F and I). In addition, calculated phonon frequency energies (*E*_LO_) of 17.5 and 16.9 meV are well consistent with the intense stretching bands of 144 and 139 cm^−1^ in Raman spectra, respectively, which further attest the strong electron–phonon interaction in deformable crystal lattice (Fig. S20). Based on the above systematical investigations, these broadband light emissions with large Stokes shifts, wide FWHM, and long lifetime can be ascribed to the radiative recombination of STEs due to the strong electron–phonon coupling arising from local deformation of crystal lattice upon photoexcitation.

**Fig. 3.**
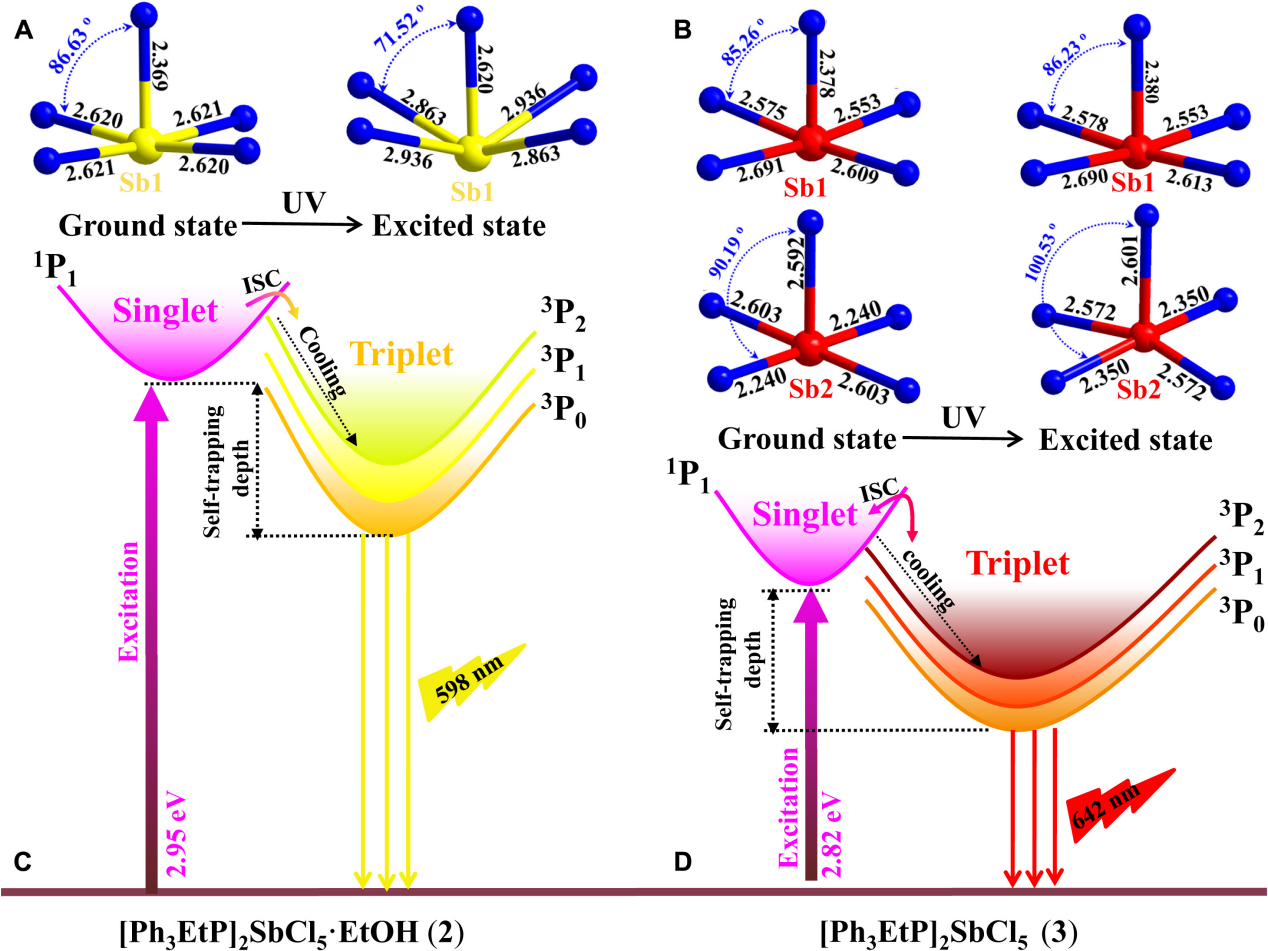
Optimized geometries of [SbCl_5_]^2−^ units at ground state and excited state for compounds 2 (A) and 3 (B), and proposed PL mechanism in configuration coordination diagram of compounds 2 (C) and 3 (D).

To confirm this hypothesis, we performed first-principles calculations on compounds **2** and **3**, simultaneously aiming to get deeper insight into the PL properties. As shown in Figs. S21 and S22, the calculated band structures give direct energy bandgaps of 2.64 and 2.56 eV, which are smaller than the experimental values from absorption spectra (2.95 and 2.82 eV) due to the limitation of software. Both the valance and conduction bands are fairly flat at ground states, indicating the absence of electronic interactions among adjacent metal halide species. The flat frontier orbitals illustrate highly localized carriers with large effective hole and electron masses, which facilitate the formation of bond excitons [49,63]. Based on the total and partial density of states, both the conduction band minimum and valence band maximum are mainly composed of Cl-3*p* and Sb-5*p* orbitals from anionic metal halide species. Although [Ph_3_EtP]^+^ participates in frontier orbitals, the strong coulomb binding leads to the localization of the carriers within anionic metal halide species [51,64]. The photo-excited electrons significantly induce the dynamic structural deformation of discrete [SbCl_5_]^2−^ units laying the foundation of lower-energy triplet STE-related broadband light emission. Subsequently, the first-principles geometry optimizations at the ground and excited state were performed to evaluate the structural deformability of anionic [SbCl_5_]^2−^ models in order to unveil their distinct PL emission efficiency [65,66]. Upon the photo-excitation, both the [SbCl_5_]^2−^ units deliver structural deformation at excited states for compounds **2** and **3** as demonstrated by the variant bond lengths and angles in Fig. 3A and B and Tables S4 and S5. For the purpose of quantitative comparison, the structural deformability is evaluated by the change of distortion degrees based on varied Sb–Cl bond lengths (Δ*d*) and Cl–Sb–Cl bond angles (*σ^2^*) based on the following equations:

$$∆d\;=\;\frac{1}{5}\sum_{n=1}^{5}{\left[\frac{d_{n}-d}{d}\right]}^{2}\;\;\sigma^{2}\;=\;\frac{1}{7}\sum_{n=1}^{8}{\left(\theta_{n}-90\right)}^{2}$$

where *d*_n_ is the individual Sb−Cl bond length and *d* represents the average bond length, and *θ*_n_ denotes the bond angle of each Cl−Sb−Cl, respectively. Remarkably, the Δ*d* and *σ^2^* values of 1.53 × 10^−3^ and 3.935 at ground state dramatically change to 3.52 × 10^−3^ and 198.169 at the excited state, indicating the higher structural deformability of discrete [SbCl_5_] unit in compound **2** (Table S6). Relatively speaking, both the [SbCl_5_]^2−^ units in compound **3** feature smaller structural deformability manifested by slightly changed geometries at excited state. Specifically, negligible structural deformation is found for [Sb(1)Cl_5_]^2−^ unit, and Δ*d* and *σ^2^* values of [Sb(2)Cl_5_]^2−^ unit change from 3.21 × 10^−3^ and 9.025 to 2.10 × 10^−3^ and 40.989 upon the photo-excitation, respectively. Obviously, the structural deformability of [SbCl_5_]^2−^ unit in compound **2** is greatly larger than those of compound **3**, giving rise to enhanced radiative recombination rate of STEs and increased PLQY from 64.47% to 91.5%. The positive relationship between structural deformability and emission efficiency adequately validates that these broadband light emissions originate from the intrinsic STEs due to strong electron–phonon coupling in deformable crystal lattice, and the detailed PL mechanism is illustrated in the configuration coordination diagram of Fig. 3C and D. Upon the photo-excitation, the electrons are excited from the ground state (^1^*S*_0_) to excited state (^1^*P*_1_) accompanied by structural deformation, resulting in the formation of STE through intersystem crossing from singlet state (^1^*P*_1_) to triplet state (^3^*P*_n_). Consequently, the radiative recombination of STE gives rise to broadband light emission with large Stokes shifts [48–52].

### SC-SC transformation with triple-mode PL switching

**Fig. 4.**
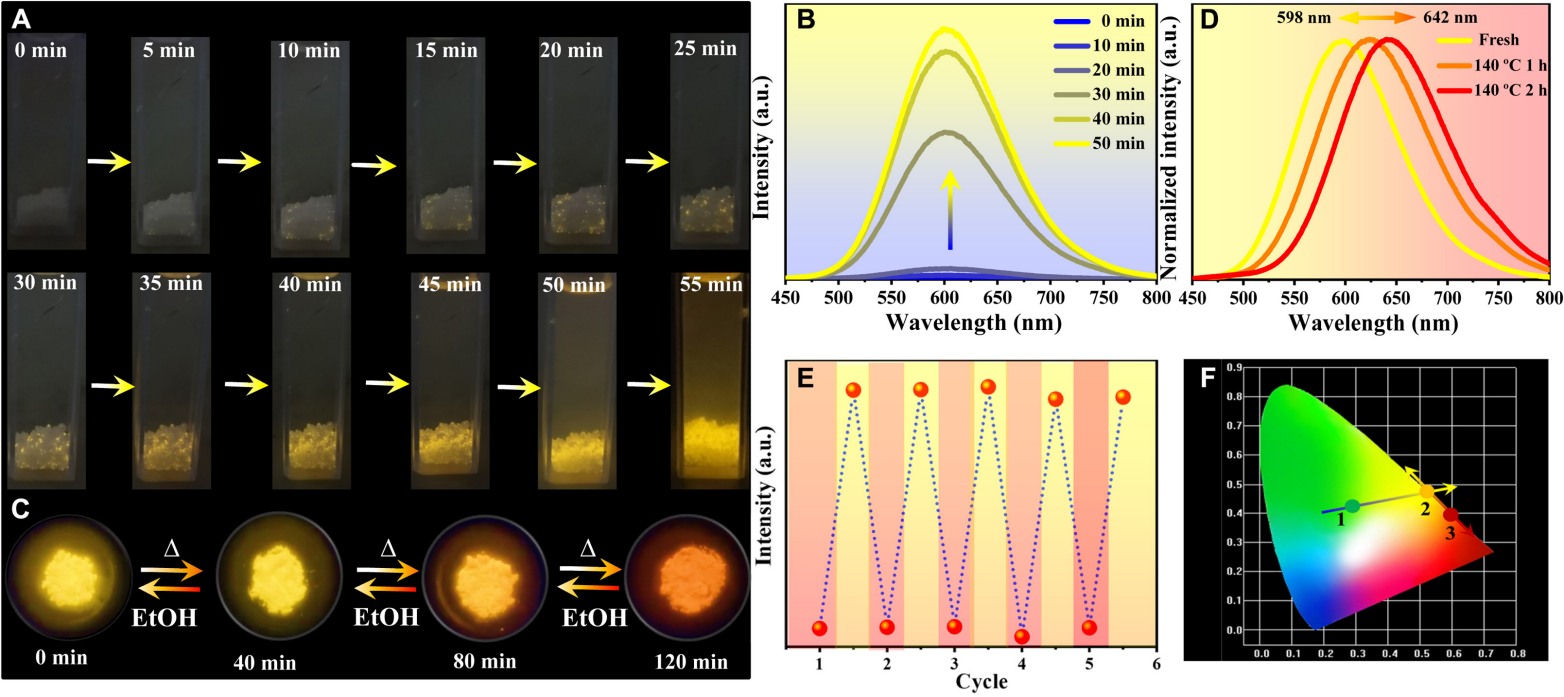
Evolution of photo-images in EtOH solvent (A) and *in situ* PL emission spectra (B) showing the PL transformation from 1 to 2; evolution of photo-images (C) and in situ PL emission spectra (D) in the reversible PL transformation between 2 and 3 under heating and EtOH trigger; the PL emission intensity during the cycle of EtOH impregnation and heating treatment as a function of cycle number (E); transition of CIE chromaticity coordinates among 1, 2, and 3 (F).

The closely related crystal structures but distinct PL performance inspire us to explore the feasible phase and PL transformation to realize stimuli-responsive PL switching between these 0D hybrid halides. Significantly, the crystal of nonluminescent compound **1** displays gradually enhanced yellow light emission once exposed to EtOH validated by the evolution of photo-images upon the 365 nm UV light irradiation (Fig. 4A). The detailed in situ PL spectral evolution also gives enhanced broadband yellow light emission centered at 598 nm, which is completely identical to that of compound **2** (Fig. 4B). The final experimental PXRD pattern after immersion in EtOH is consistent with the simulated data of compound **2**, indicating the synchronous SC-SC transformation from **1** to **2** (Fig. S23). Moreover, the XRD patterns of compound **1** and **2** after transformation were also fitted with the simulated XRD data of compounds **2** and **3** by Pawley refinement, and the fitting results gave the refinement parameters of *R*_wp_ = 7.97% and 5.54%, *R*_p_ = 5.19% and 3.58% for compounds **1** and **2** after transformation, respectively, further proving the successful structural transformations of compounds **1** and **2**. In addition, we performed X-ray single-crystal diffraction tests for the crystal after transformation to prove its SC-SC transformation. As shown in Fig. S24, the appearance of compounds **1** and **2** after the structural transformation is clearly presented on the single-crystal diffractometer with independent and strong diffraction points. The independent diffraction points, high indexation rate (96.57%), and good refinement results of obtained single-crystal **2** indicate the successful SC-SC transformation from compound **1** to **2** (Fig. S25). As a consequence, controllable turn-on switchable PL vapochromism can be realized through external stimulus-responsive SC-SC transformation, which is rarely found in 0D hybrid metal halides despite frequently reported emitting color change in this field [36,40,67–69]. Comparing with the impressible PL transformation performance of compound **1**, compound **2** displays higher structural and luminescent stability toward common organic vapor and humid air validated by the unchanged PXRD patterns and PL emission spectra after long-term exposure in these chemical environments (Figs. S26 and S27). The highly efficient and stable broadband yellow light emission endows compound **2** with potential application in solid-state white light-emitting diode (Fig. S28). However, compound **2** delivers lower thermal stability than **1** based on thermogravimetric analysis (TGA) curves mainly due to the gradual losing of guest EtOH molecule, which corresponds to the mass loss of 4.89% (Fig. S29). Interestingly, the lost of EtOH molecule in the heating process of compound **2** results in synchronous structural reassembly and SC-SC transformation to **3** accompanied by PL transformation from yellow to red color, which can also return to original yellow color after EtOH impregnation (Fig. 4C). The phase and PL transformation between **2** and **3** can be validated by the reversible evolution of PXRD patterns and *in situ* PL emission spectra (Fig. 4D and Fig. S23B). In addition, this reversible transformation exhibits excellent repeatability with almost the same emission intensity and PXRD patterns after at least 5 consecutive cycles, demonstrating fatigueless reversibility and high stability (Fig. 4E and Fig. S30). Finally, triple-mode controllable PL off–on^I^–on^II^ switching is realized in this family of 0D halide through dual external stimuli-responsive structural reassembly and stepwise SC-SC transformation, which is reported in 0D hybrid halides (Fig. 4F). In order to further explore the formation mechanism of structural transformation, the possible transformation reactions are proposed according to their chemical compositions. We then calculated the Gibbs free energy of compounds **1** to **3** and the small molecules involved to speculate on the reaction process. As shown in Table S12, the possible reaction equations have been given as Eqs. 1 to 3:

$$\begin{array}{c} {\left[{\mathrm{Ph}}_{3}\mathrm{EtP}\right]}_{2}{\mathrm{Sb}}_{2}{\mathrm{Cl}}_{8}+\text{EtOH}={\left[{\mathrm{Ph}}_{3}\mathrm{EtP}\right]}_{2}{\text{SbCl}}_{5}\cdot \text{EtOH}+{\text{SbCl}}_{3}\\ \Delta G=-4.73\times 10^{-4}\;\text{kcal}\cdot {\mathrm{mol}}^{-1} \end{array}$$ (1)

$$\begin{array}{c} {\left[{\mathrm{Ph}}_{3}\mathrm{EtP}\right]}_{2}{\text{SbCl}}_{5}\cdot \text{EtOH}={\left[{\mathrm{Ph}}_{3}\mathrm{EtP}\right]}_{2}{\text{SbCl}}_{5}+\text{EtOH}\\ \Delta G=2.53\times 10^{-5}\;\text{kcal}\cdot {\mathrm{mol}}^{-1} \end{array}$$ (2)

$$\begin{array}{c} {\left[{\mathrm{Ph}}_{3}\mathrm{EtP}\right]}_{2}{\text{SbCl}}_{5}+\text{EtOH}={\left[{\mathrm{Ph}}_{3}\mathrm{EtP}\right]}_{2}{\text{SbCl}}_{5}\cdot \text{EtOH}\\ \Delta G=-2.53\times 10^{-5}\;\text{kcal}\cdot {\mathrm{mol}}^{-1} \end{array}$$ (3)

Compound **1** can be easily and quickly reconstructed under the stimulation of EtOH to form compound **2** and a free antimony trichloride molecule is released, because the Gibbs free energy change (*ΔG*) is −4.73 × 10^−4^ kcal·mol^−1^ for this reaction. In contrast, the transition from compound **2** to **3** is relatively more difficult at room temperature, because of the positive *ΔG* of 2.53 × 10^−5^ kcal·mol^−1^. Therefore, compound **2** can only be converted to compound **3** with the additional promotion of heating treatment, which is consistent with the experimental phenomenon. Conversely, the transition from compound **3** to compound **2** becomes extremely easy with negative *ΔG* of −2.53 × 10^−5^ kcal·mol^−1^, which can occur only under the action of EtOH at room temperature. The possible reaction mechanism will help us to understand in depth the formation process of structural transformation and provide a guide to rationally design new smart PL switching in 0D hybrid metal halides.

### Application in information encryption–decryption and optical logic gate

**Fig. 5.**
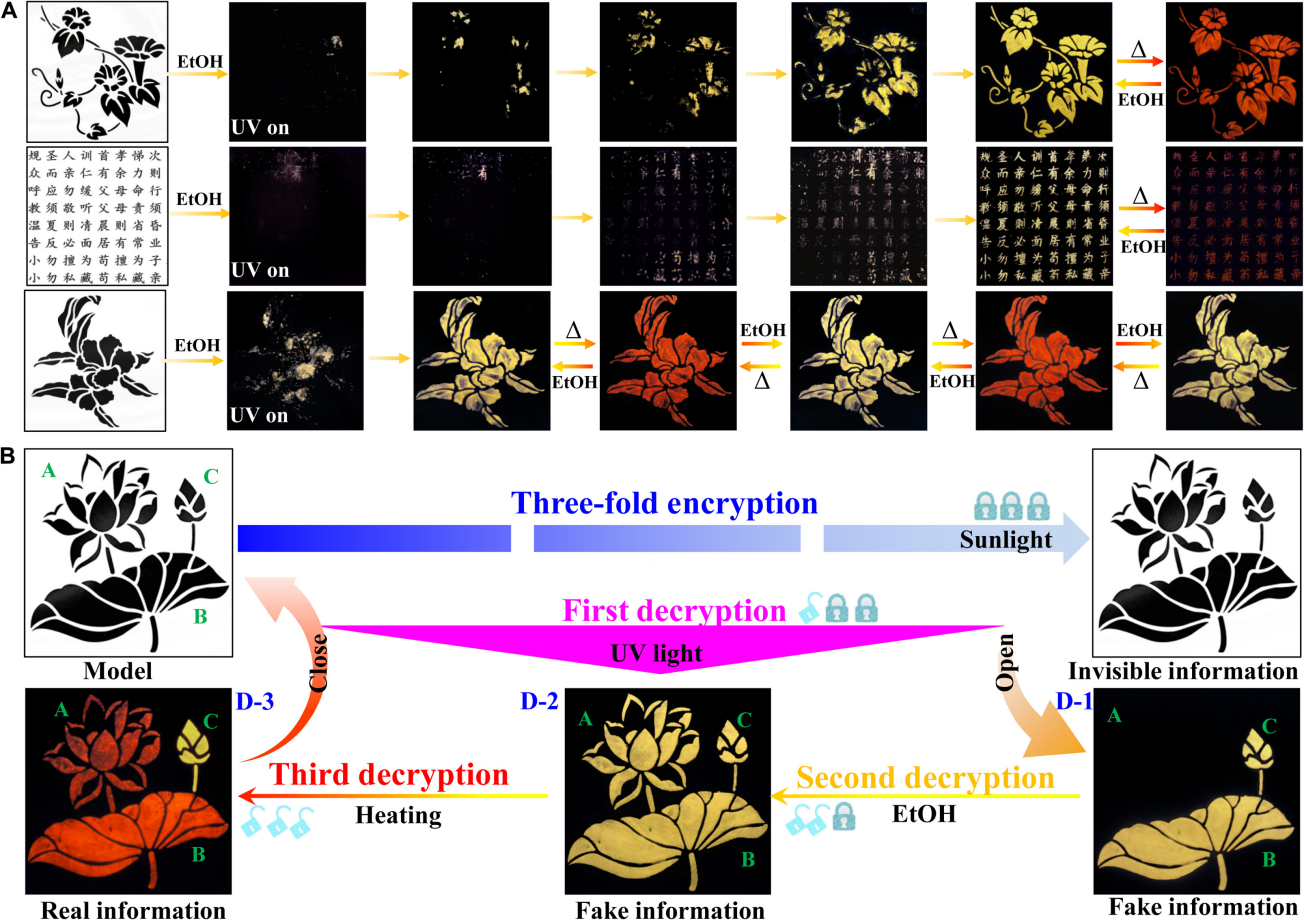
Anti-counterfeiting and information encryption–decryption applications: photo-image transitions of luminescence security patterns under 365 nm UV light irradiation (A) and schematic diagram of information encryption–decryption process in luminescence security pattern based on combined compounds 1, 2, and 3 (B).

Considering the importance of information security for commercial and military domains, the unique multiple external stimuli-responsive PL switching performance of this 0D hybrid halide family inspires us to explore the advanced applications in anti-counterfeiting and information storage as multistage security technology, after all such a three-fold mode PL switch is very rare and highly difficult to replicate (Table S2). To demonstrate the feasibility of this triplet PL switching as anti-fake technology, a series of luminescence security patterns including designed pictures and Chinese character based on compound **1** were deposited by filling powders into designed patterned grooves (Fig. 5A). All these luminescence security patterns are nearly colorless in daylight and invisible under UV light irradiation, ensuring the complete concealment of actual information. After spraying the EtOH solvent on substrates as stimuli, the invisible patterns gradually appear as yellow color with high resolution under the excitation of 365 nm UV light corresponding to SC-SC transformation from **1** to **2** (Fig. 5A). Subsequently, the yellow-emissive security patterns turn into red color after heating treatment and double anti-counterfeiting technology is successfully achieved with high security level. In addition, these luminescence security patterns also exhibit excellent repeatability and reversibility between yellow and red color with high stability. By virtue of these stimuli-responsive structural and PL transformation, single- and double-mode information encryption–decryption technologies can be achieved based on combined phases of **1**+**2** and **1**+**2**+**3** as luminescence materials, respectively, and the detailed running principles are depicted in Figs. S31 and S32. In order to enhance the decoding complexity of cryptosystem, we further propose a triplet information encryption–decryption strategy, in which only the red-emissive data were designated as correct information and others were blended as interference single. Specifically, we fabricate a combined luminescence security pattern including 3 parts of flower (A), leaf (B), and bud (C) made by **1**, **2**, and **2**@PTFE composite (PTFE = polytetrafluoroethylene), respectively (Fig. 5C). Herein, the **2**@PTFE composite is fabricated by using PTFE as protection shell to encapsulate microscale powder of **2** due to the multiple merits of high thermal stability, excellent organic solvent resistance, good transparency, and mechanical property of PTFE. As expected, the **2**@PTFE composite displays unchanged yellow light emission upon heating treatment (Fig. S33). Without the knowledge of correct decryption code, the direct excitation of UV light gives the false information revealed by B and C parts. Only under the successive EtOH trigger and heating treatment as combined decoding keys, the true information composed by A and B parts can be correctly recognized under irradiation of 365-nm UV light. These multi-fold information encryption–decryption techniques make this family of 0D halides as an ideal anti-counterfeiting platform for optical information protection and storage applications.

**Fig. 6.**
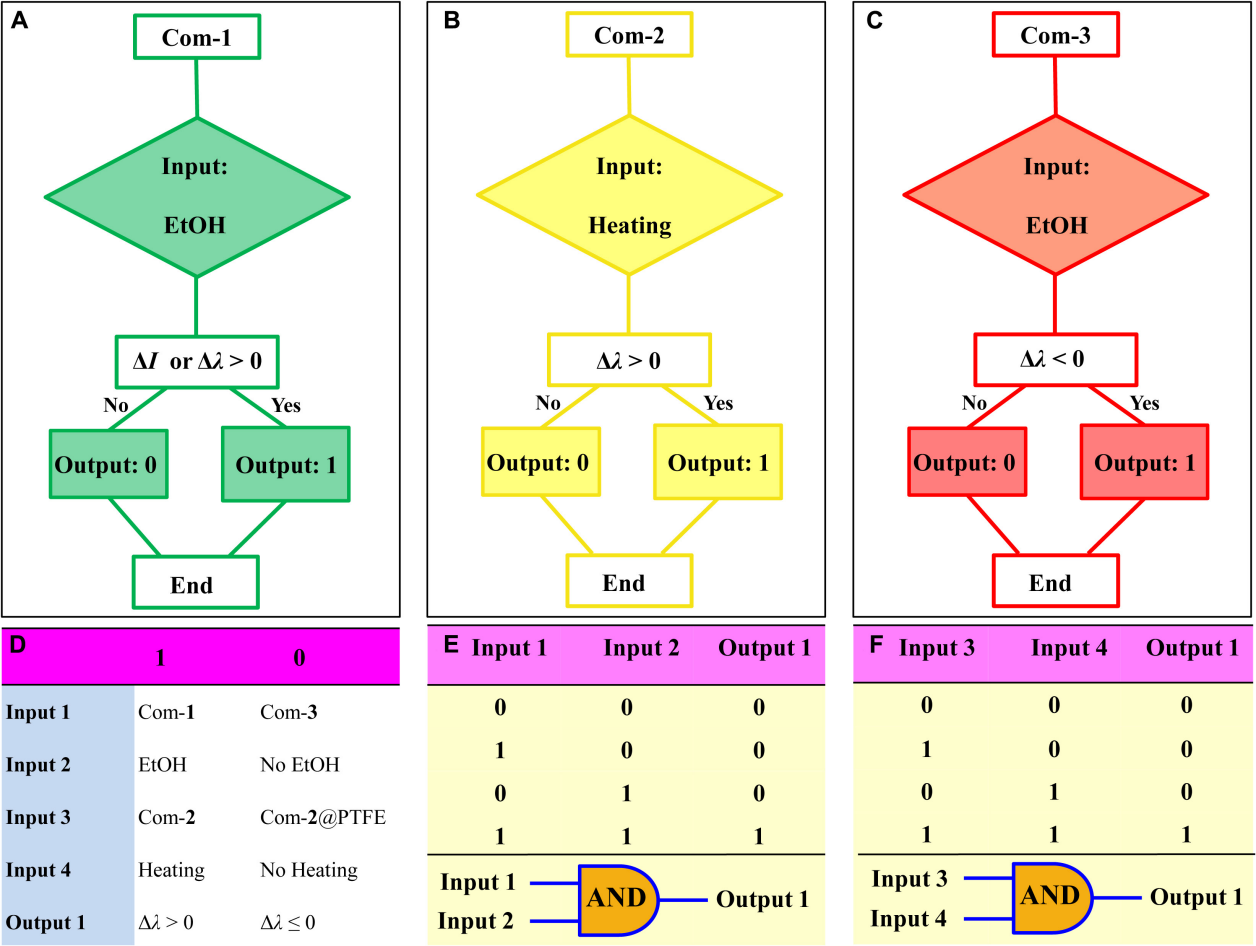
Optical logic gate applications: illustration of the binary optical logic gates based on compound 1 (A), 2 (B), and 3 (C); definition of inputs and outputs (D); and truth tables of 2-input and 2-output logic gates and schemes (E and F).

The multiple stimuli-responsive PL switching also makes this halide platform suitable for the construction of molecular-based optical logic gate used in photonic integrated circuit. Herein, the various external stimuli were defined as inputs and responsive PL switching was set as the output based on respective 0D hybrid halides. For example, we set EtOH trigger as input value and emission intensity change (Δ*I*) of halide 1 as output single (Δ*I* > 0 as “1” and Δ*I* ≤ 0 as “0”). According to the binary system, once the EtOH is impregnated as input single, the output is “1” corresponding to EtOH-induced yellow light emission; otherwise, the output is “0” representing the absence of EtOH (Fig. 6A). Similarly, by using temperature or EtOH as individual input signal and change of emission wavelength (Δ*λ*) as output value based on compound **2** or **3**, respectively, emission wavelength resolved binary optical logic gate can also be realized by virtue of the reversible PL transformation between yellow-emissive **2** and red-emissive **3** (Fig. 6B and C). In addition, by simultaneously combining the responsive PL switching of distinct luminescent materials, multicoding logic gates could be constructed based on both external stimuli and samples as dual input signals. Specifically, a pair of hybrid halides is defined as input 1 (compound **1** as “1” and compound **3** as “0”) and stimulus of EtOH is assigned as input 2 (presence as “1” and absence as “0”), and change of emission wavelength (Δ*λ*) is set as output single (Δ*λ* > 0 as “1” and Δ*λ* ≤ 0 as “0”) (Fig. 6D). As shown in the truth table of Fig. 6E, only in the case of input 1 = “1” and input 2 = “1”, the output value will be “1” and a 2-input “AND” logic gate is activated, which is related to the activated yellow light emission from compound **1** in the stimulus of EtOH. For other case, the output value will become “0” corresponding to unchanged or decreased PL emission wavelength. In a similar manner, compound **2** and **2**@PTFE composite can also be combined as input 3 representing signal of “1” and “0,” respectively, and the external temperature is defined as input 4, in which the temperature threshold is set as 100 °C (above 100 °C as “1” and below 100 °C as “0”). As a result, similar “AND” logic gate also happens for output 1 under the induction of inputs 3 and 4, where the true output of “1” is corresponding to the emission wavelength red-shift (Δ*λ* > 0) of compound **2** under the constant heating treatment (Fig. 6F). These single- and multi-input logic gates provide great potential of smart 0D hybrid halide luminescence materials in molecular recognition, sensing, information storage, keypad lock, memory devices, and so on.

## Discussion

In summary, we have demonstrated here that crystal engineering synthesized a novel family of 0D hybrid halides as a perfect structural platform to construct molecular-scale smart luminescent switching. Taking advantage of diversified anionic metal halide species as optical activity centers, three different kinds of PL performance were integrated in this 0D halide platform manifested by both distinct luminescence efficiency and emitting color. In-depth experimental and theoretical studies demonstrate that the emitting properties of 0D hybrid halides are closely related to the assembly state and configuration of metal halide blocks, which demonstrate the feasibility of local structural modulation as photon engineering strategy to dynamically regulate the PL performance. More remarkably, solvent-induced stepwise crystalline structural transition successfully results in synchronous PL transformation acting as a rare triple-model off–on^I^–on^II^ switching in 0D hybrid halide. By virtue of these external stimuli-responsive PL switching, multiple anti-counterfeiting, information encryption–decryption technologies, and dynamic optical logic gates are realized with advanced applications in information storage and security, molecular recognition, sensing, optical switch, etc. This work authenticates the effectiveness of concept about multiple external stimuli-responsive synchronous SC-SC transformation and PL switching. Such novel photon engineering strategy is expected to deepen the understanding of dynamic PL switching mechanism, guide development of new smart luminescence perovskite materials, and advance applications in cutting-edge photonic switchable device.

## Materials and Methods

### Materials

The chemical materials and regents were commercially purchased from Aladdin Chemical Company and directly used in the preparation reaction without any further purification or other physical process. SbCl_3_ (99.99%), triphenylethylphosphonium chloride ([Ph_3_EtP]Cl, 99.5%), hydrochloric acid (HCl, 37%), ethyl ether (Et_2_O), and EtOH were used.

### Synthesis of [Ph_3_EtP]_2_Sb_2_Cl_8_ (**1**)

The mixture of SbCl_3_ (1 g, 4.38 mmol) and [Ph_3_EtP]Cl (1.5 g, 4.59 mmol) was dissolved in a mixed solution of EtOH (2.5 ml) and hydrochloric acid (7.5 ml). The suspension was heated to completely dissolve and then allowed to stand at room temperature. After 1 day, lots of colorless crystals were filtrated and washed with Et_2_O three times (yield: 86% based on Sb). The structure was subsequently determined to be C_40_H_40_P_2_Sb_2_Cl_8_ by using the single-crystal X-ray diffraction. Elemental analysis (EA) calculated for C_40_H_40_P_2_Sb_2_Cl_8_: C, 43.29%; H, 3.63%; found: C, 43.32%; H, 3.68%.

### Synthesis of [Ph_3_EtP]_2_SbCl_5_·EtOH (**2**)

[Ph_3_EtP]Cl (0.287 g, 0.88 mmol) and SbCl_3_ (0.1 g, 0.44 mmol) were dissolved in a mixed solution of EtOH (4 ml) and hydrochloric acid (0.5 ml). The suspension was constantly transferred into a 20 ml glass vial, which was then sealed and heated to a constant temperature of 80 °C for 5 days. After the reaction, lots of colorless block crystals were filtrated from the vial and washed with EtOH three times (yield: 78% based on Sb). The structure was subsequently determined to be C_42_H_45_OP_2_SbCl_5_ by using the single-crystal X-ray diffraction. EA calculated for C_42_H_45_OP_2_SbCl_5_: C, 54.43%; H, 4.89%; found: C, 54.42%; H, 4.87%.

### Synthesis of [Ph_3_EtP]_2_SbCl_5_ (**3**)

Compound **2** powder samples (0.2 g) were placed in a glass bottle and then heated to 140 °C under vacuum conditions for 2 h. After slow cooling to room temperature, lots of colorless block crystals were obtained (yield: 92% base on compound **2**). The structure was subsequently determined to be C_40_H_40_P_2_SbCl_5_ by using the single-crystal X-ray diffraction. EA is calculated for C_40_H_40_P_2_SbCl_5_: C, 54.49%; H, 4.57%; found: C, 54.50%; H, 4.54%.

### Synthesis of **2**@PTFE

Compound **2** powders (0.5 g) were dispersed into 3 ml of EtOH to obtain a homogeneous suspension. Then, 0.1 ml of PTFE solution was placed in the solution and stirred at room temperature for 8 h. White powder of **2**@PTFE was obtained by evaporation solvent.

### Characterizations

The PXRD of compounds **1** to **3** were performed on a Rigaku Dmax 2500 and Rigaku SmartLab (Dtex250, Japan) X-ray powder diffraction meter equipped with Cu-*Kα* radiation (λ = 1.5418 Å). EA of C, H, and N was carried out on elemental analyzer of Vario EL-Cube. The solid-state UV–Vis optical absorption spectrum were performed on a PE Lambda 900 UV/Vis spectrophotometer. The thermal stabilities were tested by using the TGA, which were carried out on a Netzsch STA449C thermal analyzer in the temperature range of 25 to 800 °C under the constant protection of N_2_ atmosphere flow with a heating rate of 10 °C·min^−1^. The Raman measurements were performed by using Horiba Scientific LabRam HR Evolution under 532-nm excitation wavelength. SEM images were obtained by Zeiss Sigma 500. The PL spectra, time-resolved decay data, and PLQYs were performed on an Edinbergh FLS1000 fluorescence spectrometer.

## Data Availability

All data that support the findings of this study are available from the corresponding author upon reasonable request.

## Supplementary Materials

Supplementary 1 Experimental Procedures Figs. S1 to S33 Tables S1 to S12
