# Supplementary material for: Stepwise Crystalline Structural Transformation in 0D Hybrid Antimony Halides with Triplet Turn-on and Color-Adjustable Luminescence Switching
Source: Research (Wash D C). 2023 Mar 30;6:0094. doi: 10.34133/research.0094 (PMC10062499; doi:10.34133/research.0094)
Supplement: Supplementary 1 — Experimental Procedures Figs. S1 to S33 Tables S1 to S12 [file research.0094.f1.doc]

**Supplementary Material**

**Stepwise Crystalline Structural Transformation in 0D Hybrid Antimony Halides with Triplet Turn-on and Color-Adjustable Luminescence Switching**

Jian-Qiang Zhao,^1^ Yue-Yu Ma,^1^ Xue-Jie Zhao,^1^ Yu-Jia Gao,^1^ Zi-Yan Xu,^1^ Pan-Chao Xiao,^1^ Cheng-Yang Yue,^1^* and Xiao-Wu Lei^1^*

School of Chemistry, Chemical Engineer and Materials, Jining University, Qufu, Shandong, 273155, P. R. China.

Correspondence should be addressed to Xiao-Wu Lei; xwlei_jnu@163.com and Cheng-Yang Yue; yuechengyang@126.com

| **Table of Contents** | |
| --- | --- |
| **Figure S1** | The stacking structure of compound **1** along *a*-axis. |
| **Figure S2** | The stacking structure of compound **2** along *c*-axis. |
| **Figure S3** | The stacking structure of compound **3**. |
| **Figure S4** | The experimental and simulate PXRD patterns of compounds **1**-**3** (a-c). |
| **Figure S5** | Elemental mapping images of compounds **1**-**3** (a-c). |
| **Figure S6** | The UV-Vis absorption spectra and their corresponding photo energy of compounds **1** (a, c), **2** (b, d) and **3** (c, e) based on Tauc plots. |
| **Figure S7** | The PL decay curve monitoring at 530 nm emission for compound **1** at 300 K. |
| **Figure S8** | PLQY of compounds **2** (a) and **3** (b) at 300 K. |
| **Figure S9** | The emission wavelength dependent PL excitation spectra of compound **2**. |
| **Figure S10** | The excited wavelength dependent PL emission spectra of compound **3** from 200-400 nm. |
| **Figure S11** | The emission wavelength dependent PL excitation spectra of compound **3** at 300 K. |
| **Figure S12** | The consecutive 3D PL excitation and emission correlation maps of compound **3** at 300 K. |
| **Figure S13** | SEM photo images of bulk (a) and microscale (b) crystals of compound **2**. |
| **Figure S14** | SEM photo images of bulk (a) and microscale (b) crystals of compound **3**. |
| **Figure S15** | Comparisons of PL emission spectra of bulk crystals and microscale powders for compound **2**. |
| **Figure S16** | Comparisons of PL emission spectra of bulk crystals and microscale powders for compound **3**. |
| **Figure S17** | The PL emission intensity versus excitation power for the bulk crystals of compound **3**. |
| **Figure S18** | Temperature dependent PL emission spectra of compound **2**. |
| **Figure S19** | Temperature dependent PL emission spectra (a), the temperature-dependent emission energy evolution (green line) with the contribution of TE (red line) and EP interactions (blue line) (b), and the temperature dependence of emission intensity (c) of compound **3**. |
| **Figure S20** | Raman spectra of compounds **2** and **3** excited by 532 nm laser. |
| **Figure S21** | The calculated band structures and density of states for ground state (a-b) and excited state (c-d) of compound **2**. |
| **Figure S22** | The calculated band structures and density of states for ground state (a-b) and excited state (c-d) of compound **3**. |
| **Figure S23** | (a) PXRD patterns of compound **1** after transformation, simulated date of compound **2**, their difference plot, and the positions of the Bragg reflections, (b) PXRD patterns of compound **2** after transformation, simulated date of compound **3**, their difference plot, and the positions of the Bragg reflections. |
| **Figure S24** | The single crystal photos of compound **1** (a) and **2** (b) after the structural transformation on the single-crystal diffractometer. |
| **Figure S25** | X-ray single crystal diffraction experiment after the transformation. |
| **Figure S26** | Comparisons of experimental PXRD patterns (a) and PL emission spectra (b) of compound **2** before and after exposure in humid air for 100 days. |
| **Figure S27** | The PXRD patterns and PL emission spectra of compound **2** after soaking various organic solvents over one day. |
| **Figure S28** | Characterizations of white LED fabricated by mixing the yellow phosphor compound **2** and blue phosphor BaMgAl_10_O_17_:Eu^2+^ on a UV LED chip. |
| **Figure S29** | The thermogravimetric analysis (TGA) curves of compound **1** (a), compound **2** (b) and compound **3** (c). |
| **Figure S30** | The experimental PXRD pattern and simulated date of compound **3** after reversible transformation between compounds **2** and **3** over 5 cycles. |
| **Figure S31** | Illustration of single-mode digital information encryption-decryption process based on compounds **1** to **2**. |
| **Figure S32** | Illustration of double-mode digital information encryption-decryption process based on compounds **1** to **3**. |
| **Figure S33** | Comparison of the PL spectra of **2**@PTFE composite before and after heating treatment. |
| **Table S1** | Summary of the PL properties of single crystalline antimony perovskites at 300 K. |
| **Table S2** | Summary of the applications for anti-counterfeiting, information encryption-decryption and optical logic gates of 0D antimony halide perovskites bulk crystals. |
| **Table S3** | Crystal Data and Structural Refinements for [Ph_3_EtP]_2_Sb_2_Cl_8_ (**1**), [Ph_3_EtP]_2_SbCl_5_·EtOH (**2**) and [Ph_3_EtP]_2_SbCl_5_ (**3**). |
| **Table S4** | Selected bond lengths (Å) and bond angles (°) for compound **1**. |
| **Table S5** | Comparison of the bond length and angles in [SbCl_5_]^2-^ square pyramid at ground and excited states of [Ph_3_EtP]_2_SbCl_5_·EtOH (**2**). |
| **Table S6** | Comparison of the bond length and angles in [SbCl_5_]^2-^ square pyramid at ground and excited states of [Ph_3_EtP]_2_SbCl_5_ (**3**). |
| **Table S7** | Comparison of the distortion degrees of [SbCl_5_]^2-^ square pyramid at ground and excited states of [Ph_3_EtP]_2_SbCl_5_·EtOH (**2**) and [Ph_3_EtP]_2_SbCl_5_ (**3**). |
| **Table S8** | Hydrogen bonds data for compound **1**. |
| **Table S9** | Hydrogen bonds data for compound **2**. |
| **Table S10** | Hydrogen bonds data for compound **3**. |
| **Table S11** | Crystal data and structure refinement of compound **1** after transformation. |
| **Table S12** | Calculated energy for compounds **1**-**3**, EtOH and SbCl_3_. |

**Experimental Procedures**

***Single crystal X-ray diffraction*:** The single crystal data of compound **1** was collected on XtaLAB Synergy, Dualflex, HyPix diffractometer with Cu *Kα* (*λ* = 1.54184 Å) radiation at 100 K. The single crystal data of compound **2** was collected on the Bruker Apex II CCD diffractometer with Mo *Kα* radiation (*λ* = 0.71073 Å) at room temperature. The single crystal data of compound **3** was collected on ROD, Synergy Custom system, HyPix diffractometer with Ga *Kα* (*λ* = 1.34050 Å) radiation at 100 K. These crystal structures were solved and refined by full matrixcs methods against *F*^2^ using SHELXL-2014 program package and Olex-2 software.^[1]^ All the non-hydrogen atoms were refined with anisotropic thermal parameters, and hydrogen atoms of organic molecules were positioned geometrically and refined isotropically. Structural refinement parameters of compounds **1**-**3** were summarized in Table S3 and important bond lengths and angles are listed in Table S4-10. CCDC (number of 2154381-2154383) contains the supplementary crystallographic data for this paper.

***Photoluminescence property characterizations*:** The PL spectra were performed on an Edinbergh FLS1000 fluorescence spectrometer. The time-resolved decay data were carried out using the Edinburgh FLS1000 fluorescence spectrometer with a picosecond pulsed diode laser. The average lifetimes were obtained by exponential fitting. The PLQY measurements were performed on an Edinburgh FLS1000 steady state/transient state fluorescence spectrometer with an integrating sphere (BaSO_4_ coating as reference material) using single photon counting mode. Emission from the sample were guided through a single grating Czerny-Turner monochromator and detected by a Hamamatsu R928P photomultiplier tube. The PLQYs were calculated based on the equation: *ƞ*_QE_ = *I*_S_/(*E*_R_**-***E*_S_), which *I*_S_ represents the luminescence emission spectrum of the sample, *E*_R_ is the spectrum of the excitation light from the empty integrated sphere (BaSO_4_ coating), and *E*_S_ is the excitation spectrum for exciting the sample. The power-dependent PL spectra were measured using the 375 nm (LE-LS-375-140TFCA, 1-140 mW). The CIE chromaticity coordinates, and CRI were calculated using the CIE calculator software based on the emission spectrum.

The thermal activation energy can be calculated by using the Arrhenius-type formula:

$$I\text{PL}=\frac{I\text{0}}{1+a\exp\left( \frac{-E\text{a}}{k\text{B}T} \right)}$$

where *I*_PL_ was the emission intensity at different temperature (*T*), *I*_0_ was the integrated emission intensity at 0 K, *E*_a_ belongs to the activation energy related to the thermally activated non-radiation process, and *k*_B_ represented the Boltzmann constant.

***Fabrication of WLED lamp*:** The WLED lamp device was fabricated by combining a 365 nm UV LED chip with the yellow phosphor of **2** and the commercial BaMgAl_10_O_17_:Eu^2+^ blue phosphor. Yellow phosphor of **2** and blue phosphor BaMgAl_10_O_17_:Eu^2+^ were mixed with epoxy resin and stirred continuously for 10 min. Then the mixture was coated on the surface of the UV LED chip and cured for 30 min under vacuum conditions. The optical properties of the fabricated WLEDs were evaluated by a temperature-programmed LED optoelectronic analyzer with an integrating sphere (EVERFINE HAAS-2000).

***Theoretical calculation*:** The single crystal data of compounds **2**-**3** were directly used to calculate the electronic band structure in Castep software.^[2]^ The total energy was calculated with density functional theory (DFT) using Perdew-Burke-Ernzerhof (PBE) generalized gradient approximation. Hence, the C-2*s*^2^2*p*^2^, P-3*s*^2^3*p*^3^, H-1*s*^1^, Sb-5*s*^2^5*p*^3^ and Cl-3*s*^2^s*p*^5^ orbital were adopted as valence electrons. The number of plane wave included in the basis sets was determined by a cutoff energy of 320 eV and numerical integration of the Brillouin zone is performed using Monkhorst-Pack k-point sampling of 2×2×2. The excited-state structures were calculated by the VASP to perform all the DFT calculations within the generalized gradient approximation (GGA) using the PBE formulation. ^[3-5]^ Following Franck-Condon principle, the optical excitation and emission energies were obtained by calculating the total energy differences between the excited and the ground states using PBE0-optimized ground-state and excited-state structures, respectively. The geometry optimizations were considered convergent when the force changes were smaller than 0.03 eV/Å. The calculations of energy were performed by using the Gaussian 16 software.^[6]^ The crystal structure of compounds **1**-**3** and other small molecule were fully optimized by DFT using the B3LYP functional with the SDD basis set for Antimony and the 6-311G* basis set for the other elements. For the optimized structures, harmonic vibrational frequencies (all real) were calculated to confirm that all the optimized structures correspond to energy minima.

***Anti-counterfeiting, information encryption and decryption applications.*** The anti-counterfeiting and information encryption-decryption printing experiments were performed on the hollow materials with specific patterns and the nylon plastic board with grooves. Compounds **1**-**3** samples were evenly filled to the hollow materials and the nylon plastic board, respectively. The stepwise PL transformation were induced by dripping ethanol or heating to achieve multiple information encryption-decryption and fluorescent switches.

**
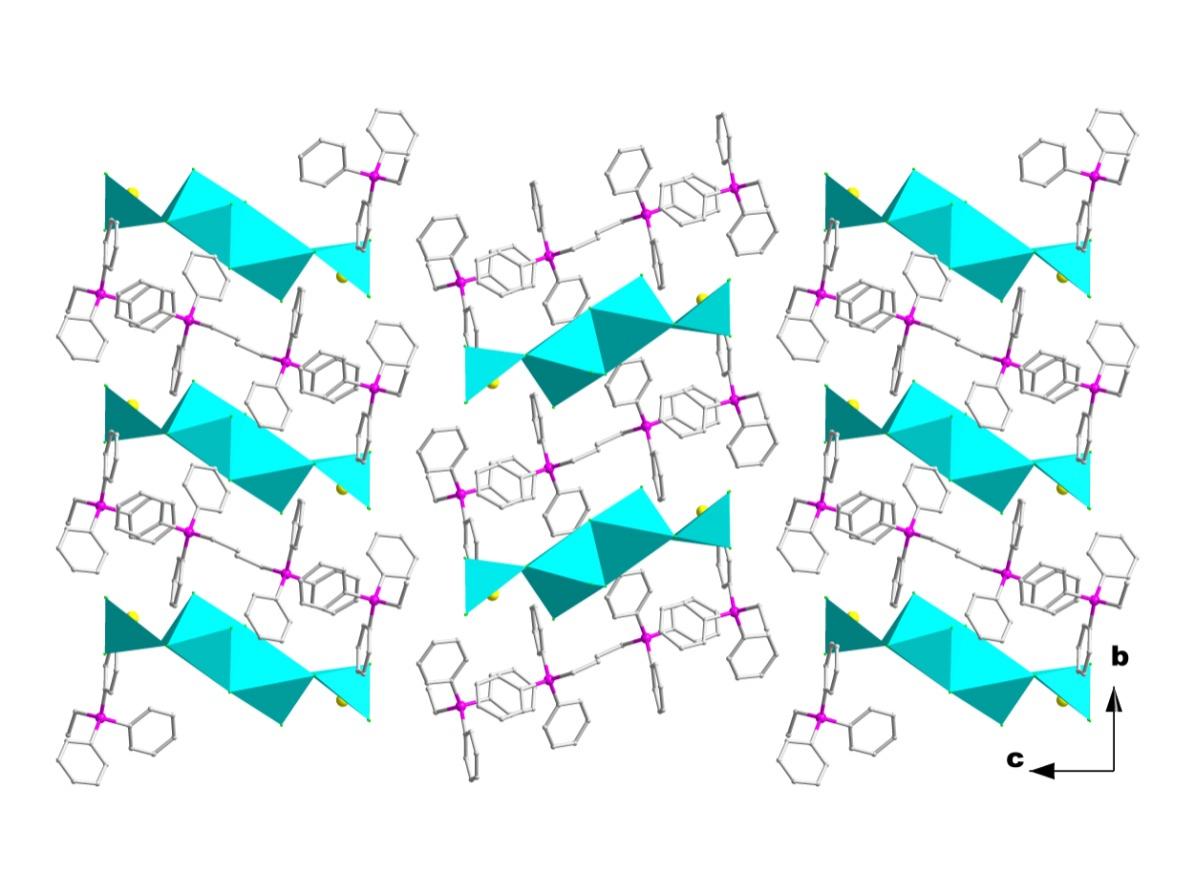
**

**Figure S1.** The stacking structure of compound **1** along *a*-axis.


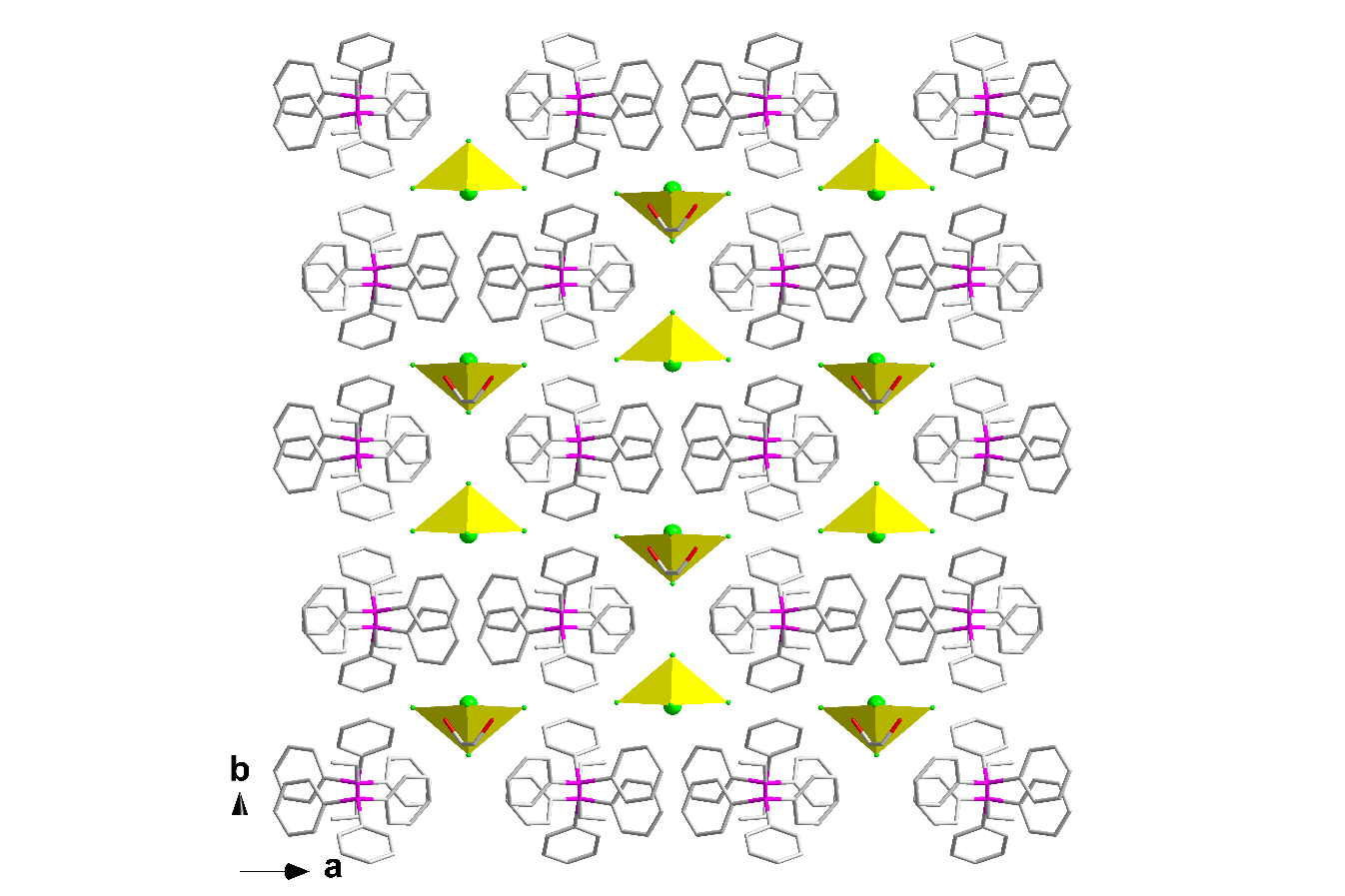


**Figure S2**. The stacking structure of compound **2** along *c*-axis.

**
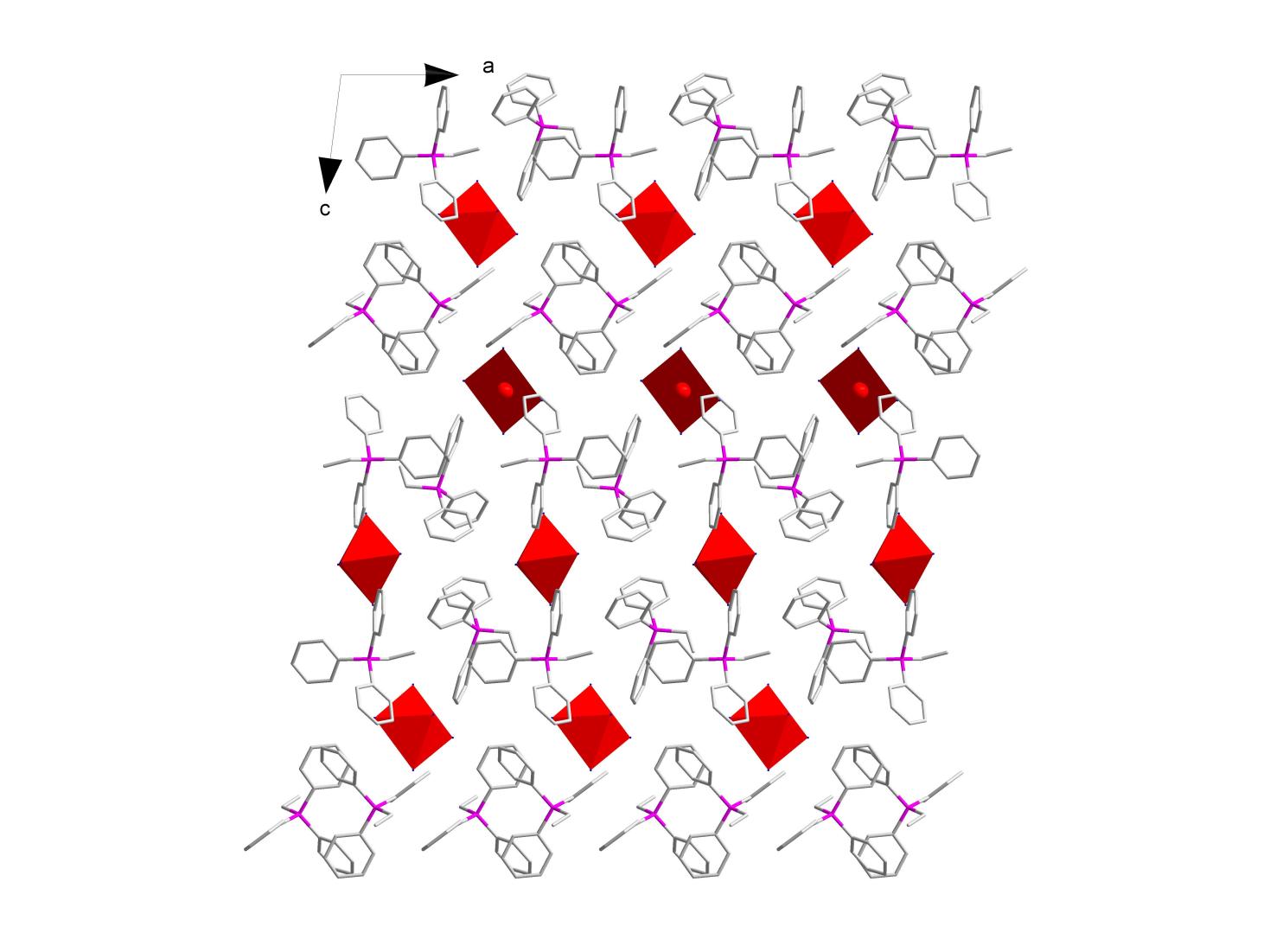
**

**Figure S3**. The stacking structure of compound **3** along *b*-axis.

****(a)

****(b) ****(c)

**Figure S4.** The experimental and simulate PXRD patterns of compounds **1**-**3** (a-c).


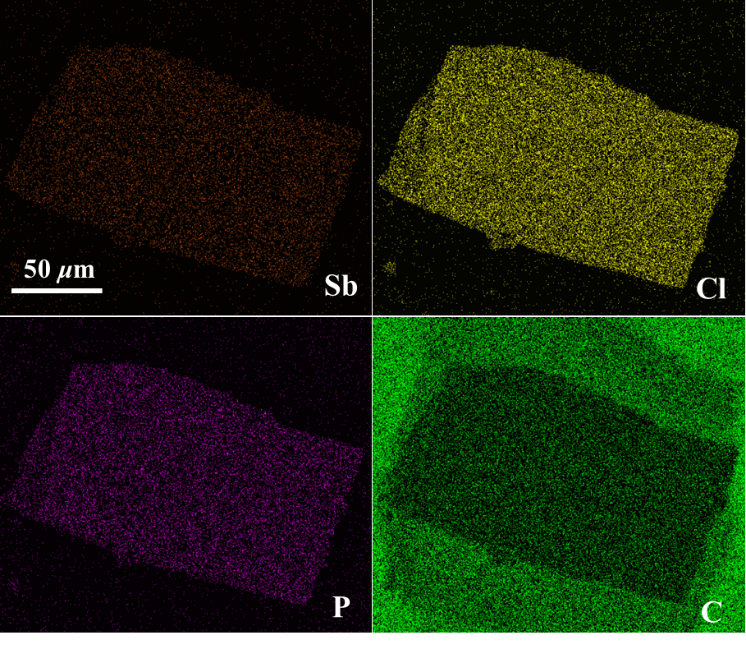
(a)


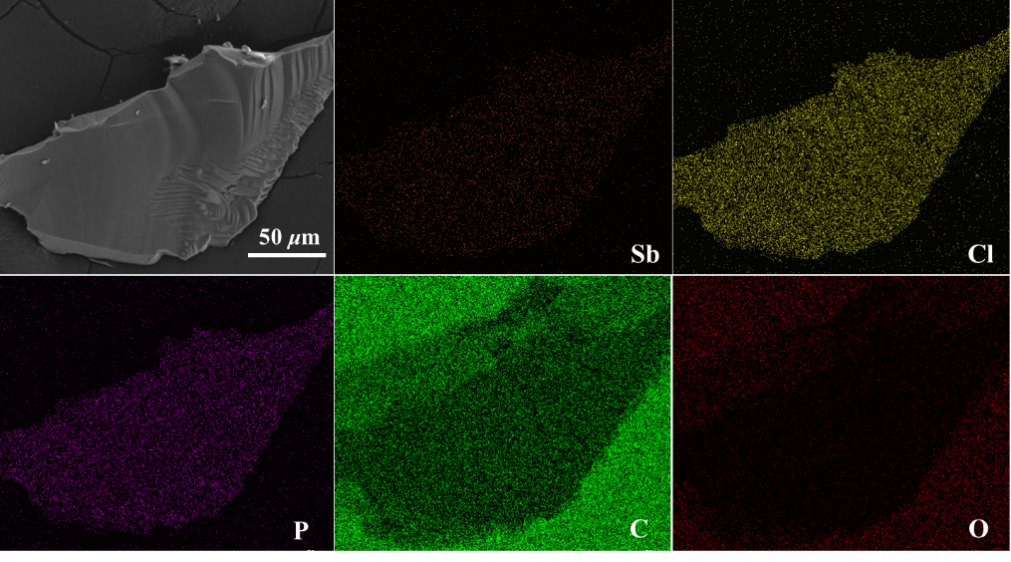
(b)


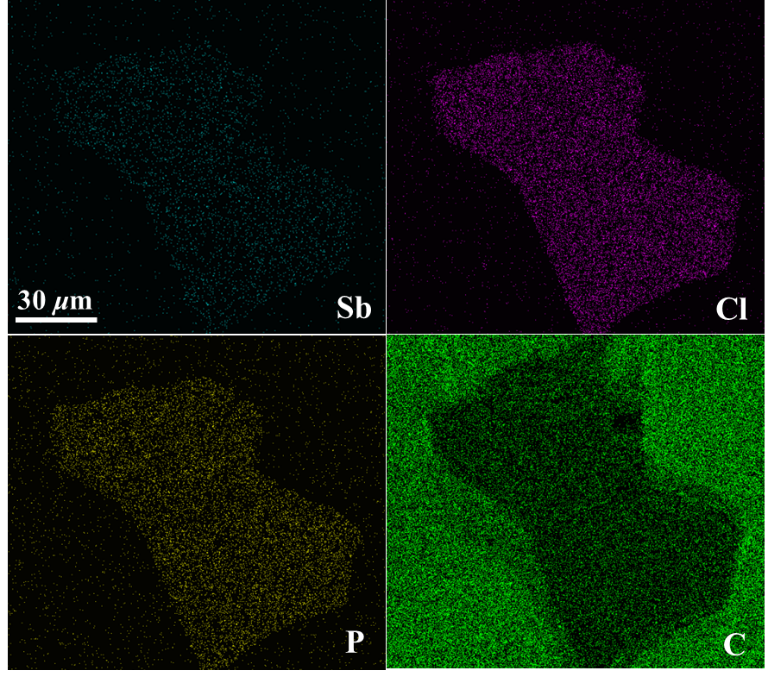
(c)

**Figure S5.** Elemental mapping images of compounds **1**-**3** (a-c).

**
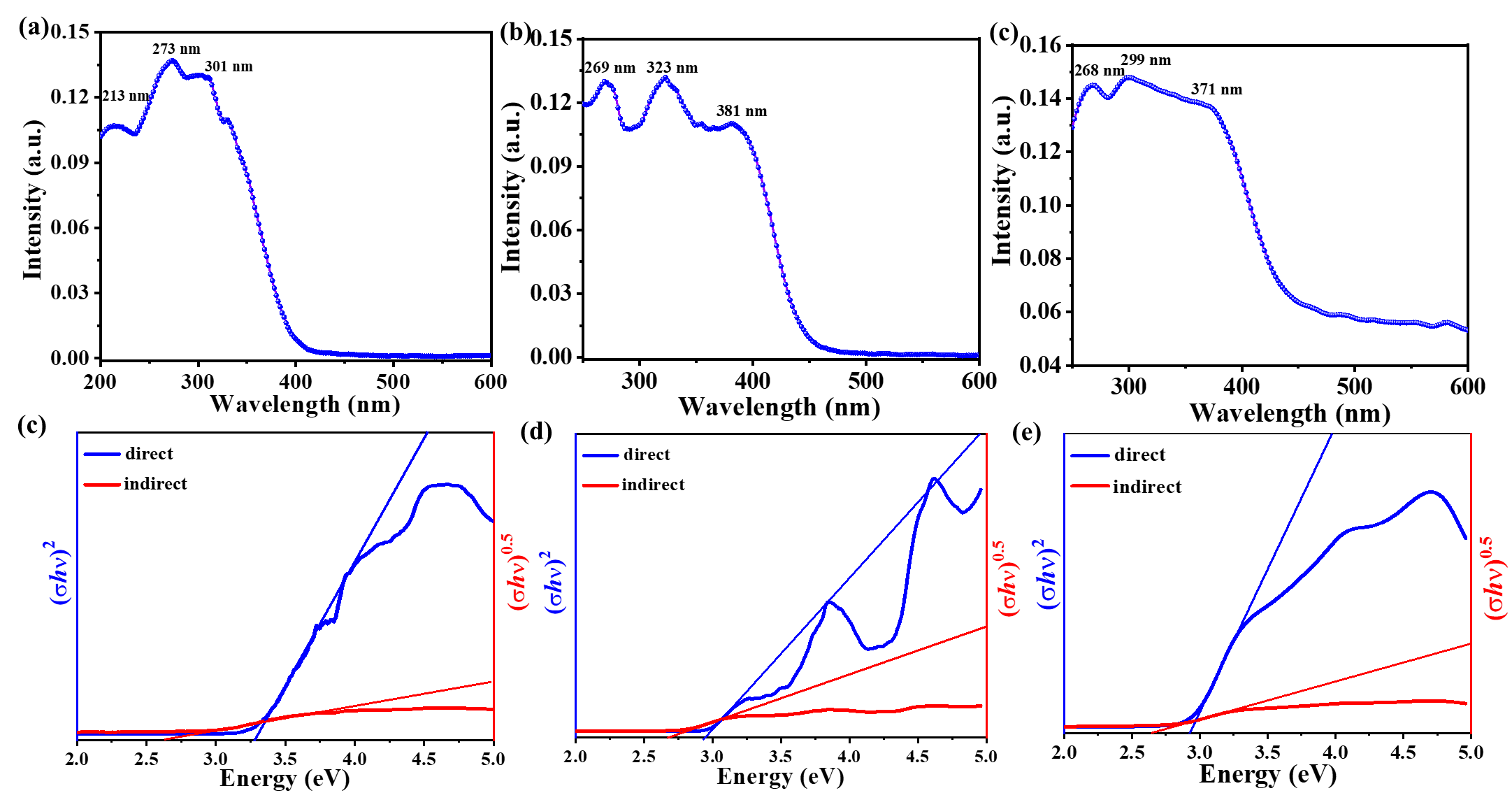
**

**Figure S6.** The UV-Vis absorption spectra and their corresponding photo energy of compounds **1** (a, c), **2** (b, d) and **3** (c, e) based on Tauc plots.

**Figure S7.** The PL decay curve monitoring at 530 nm emission for compound **1** at 300 K.

****(a)

****(b)

**Figure S8.** PLQY of compounds **2** (a) and **3** (b) at 300 K.

**Figure S9.** The emission wavelength dependent PL excitation spectra of compound **2**.

****(a)

****(b)

****(c)

**Figure S10.** The excited wavelength dependent PL emission spectra of compound **3** from 200-400 nm at 300 K.

****(a)

****(b)

****(c)

**Figure S11.** The emission wavelength dependent PL excitation spectra of compound **3** at 300 K.

**Figure S12.** The consecutive 3D PL excitation and emission correlation maps of compound **3** at 300 K.


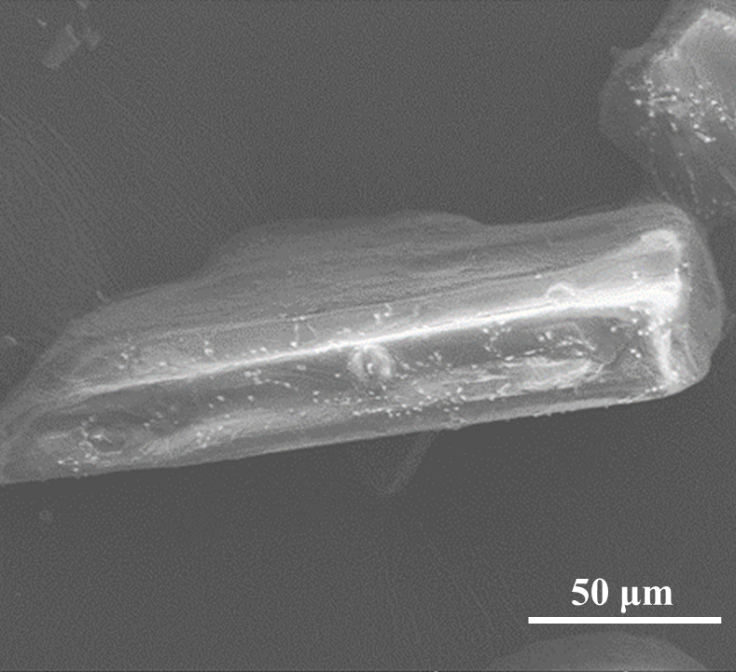
(a)

**
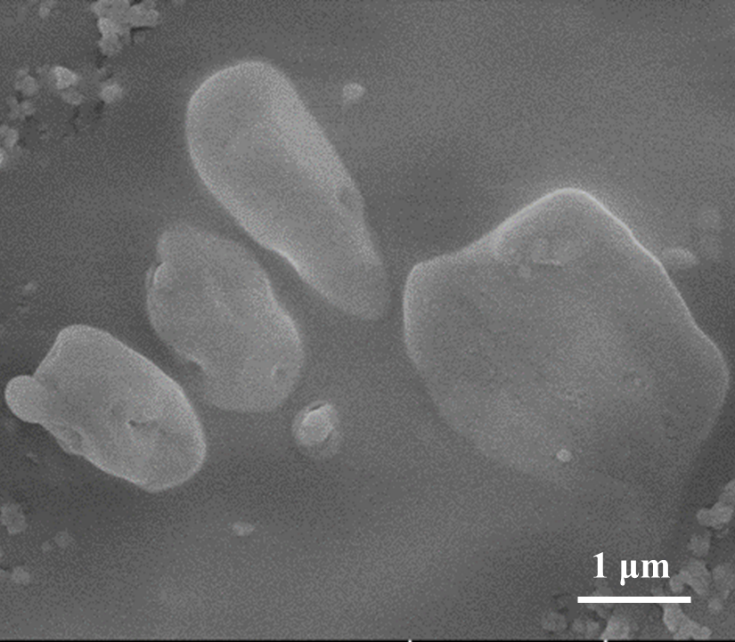
**(b)

**Figure S13.** SEM photo images of bulk (a) and microscale (b) crystals of compound **2**.


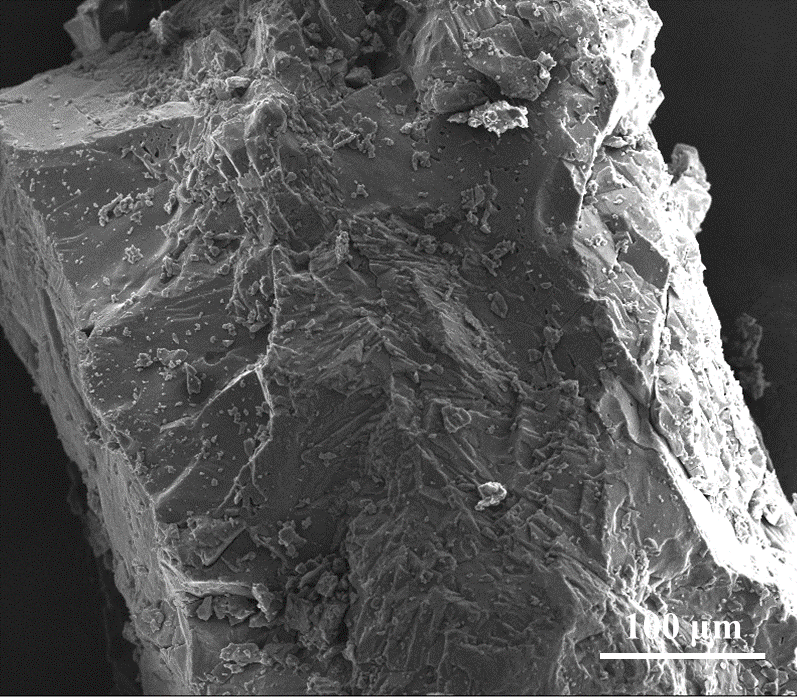
(a)

**
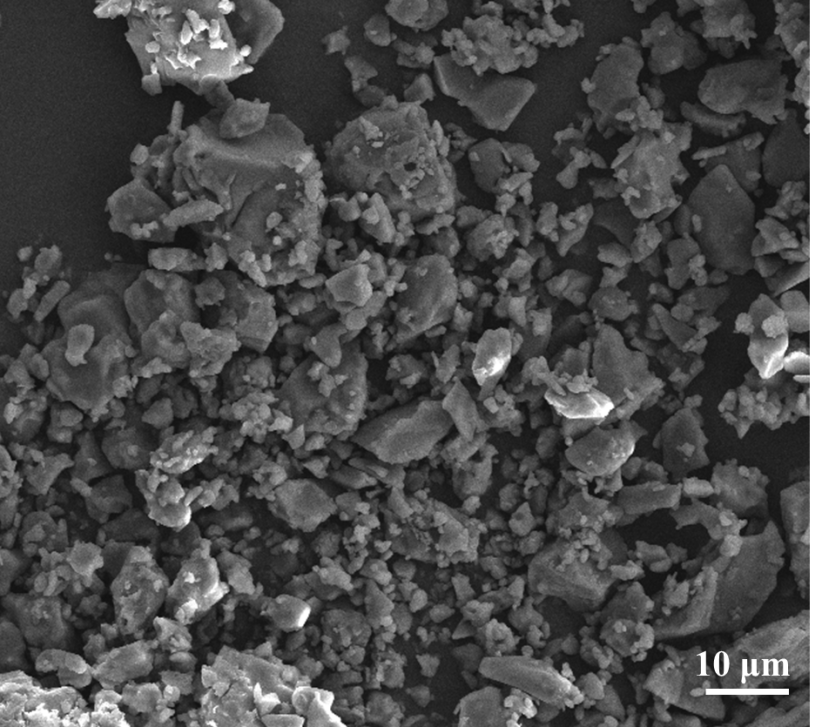
**(b)

**Figure S14.** SEM photo images of bulk (a) and microscale (b) crystals of compound **3**.

**Figure S15.** Comparisons of PL emission spectra of bulk crystals and microscale powders for compound **2**.

(b)

**Figure S16.** Comparisons of PL emission spectra of bulk crystals and microscale powders for compound and **3**.

**Figure S17.** The PL emission intensity versus excitation power for the bulk crystals of compound **3**.

**Figure S18.** Temperature dependent PL emission spectra of compound **2**.

(a)

(b)

(c)

**Figure S19.** Temperature dependent PL emission spectra (a), the temperature-dependent emission energy evolution (green line) with the contribution of TE (red line) and EP interactions (blue line) (b), and the temperature dependence of emission intensity (c) of compound **3**.

(a)(b)

**Figure S20.** Raman spectra of compounds **2** and **3** excited by 532 nm laser.


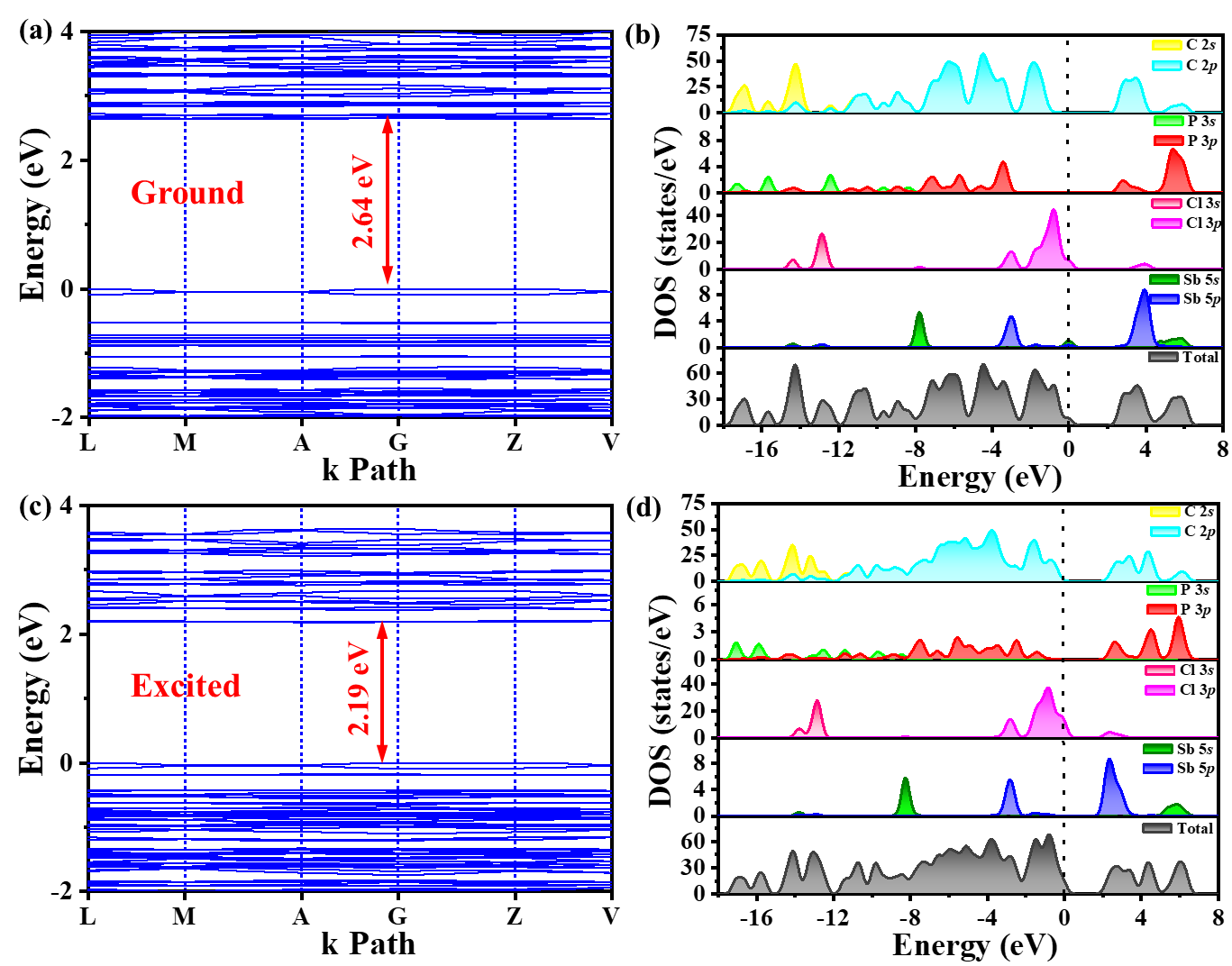


**Figure S21.** The calculated band structures and density of states for ground state (a-b) and excited state (c-d) of compound **2**.


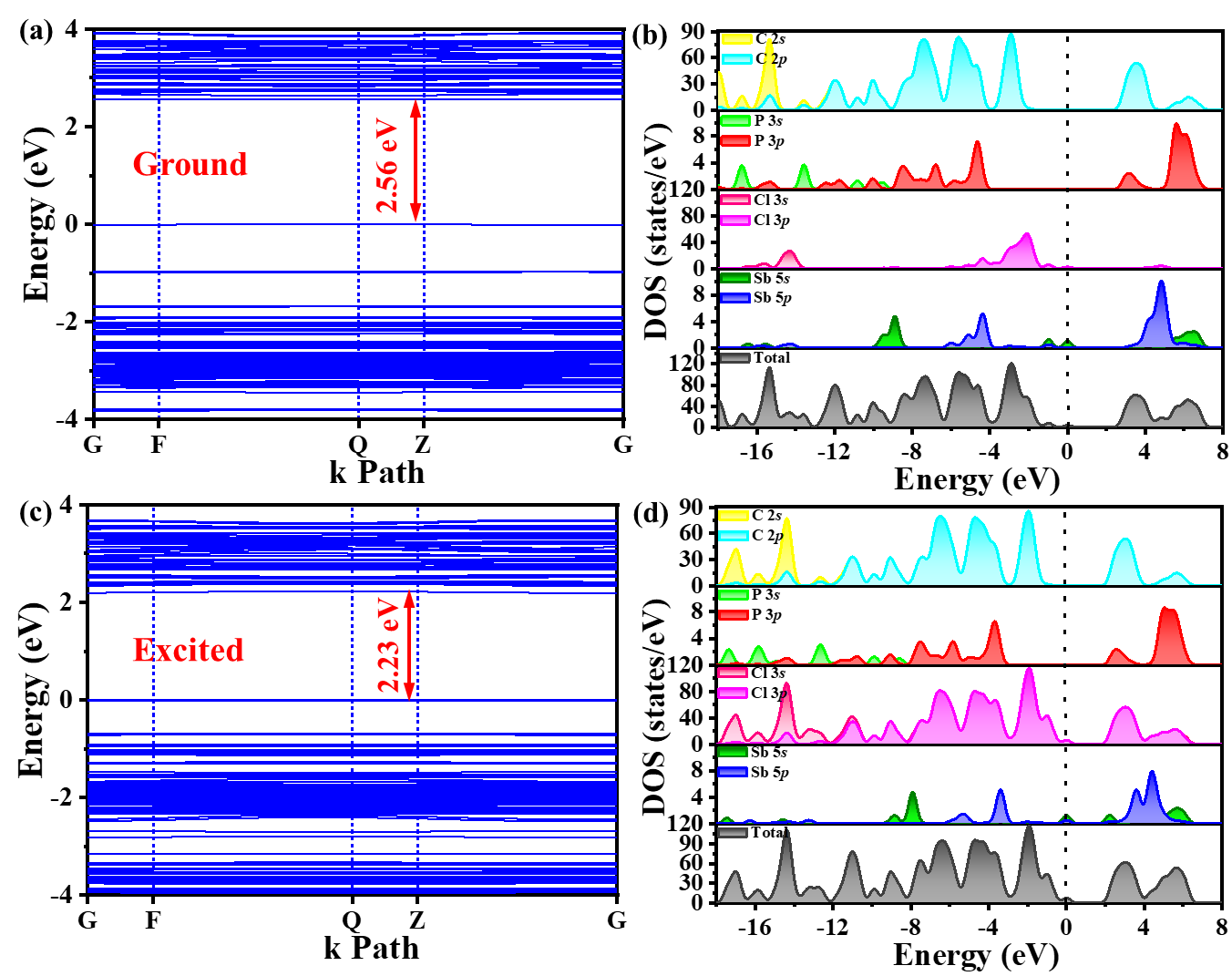


**Figure S22.** The calculated band structures and density of states for ground state (a-b) and excited state (c-d) of compound **3**.

**(a)

**(b)

**Figure S23.** (a) PXRD patterns of compound **1** after transformation, simulated date of compound **2**, their difference plot, and the positions of the Bragg reflections, (b) PXRD patterns of compound **2** after transformation, simulated date of compound **3**, their difference plot, and the positions of the Bragg reflections.


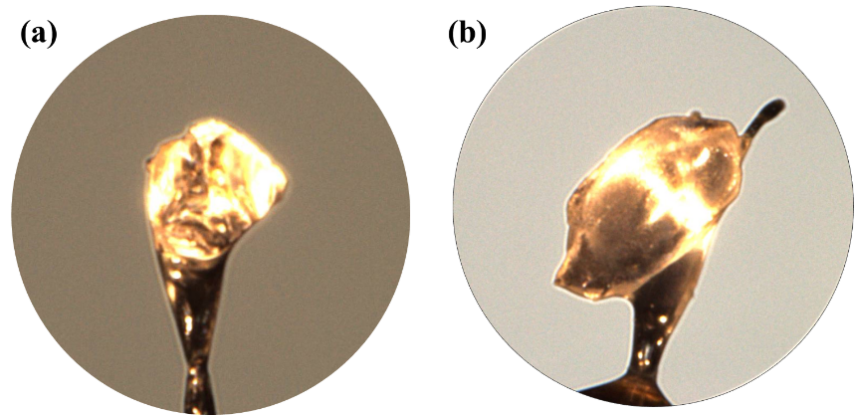


**Figure S24.** The single crystal photos of compound **1** (a) and **2** (b) after the structural transformation on the single-crystal diffractometer.


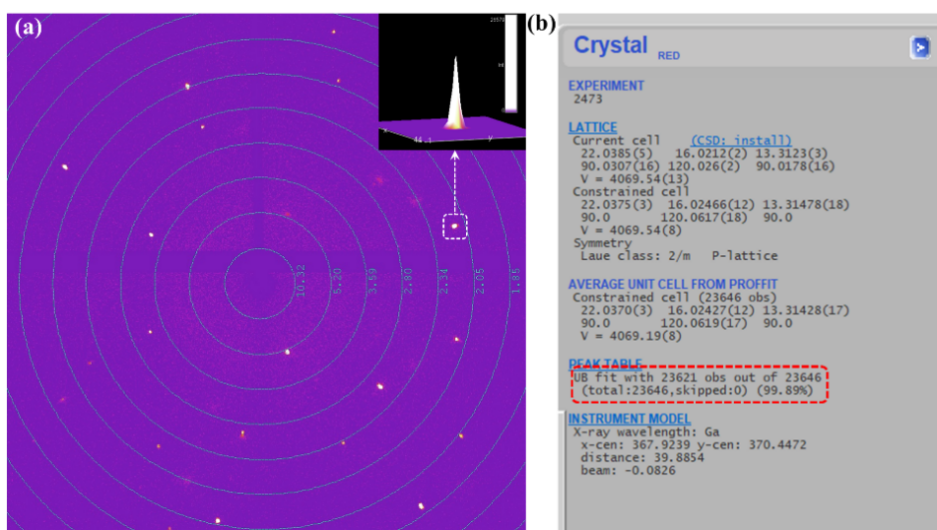


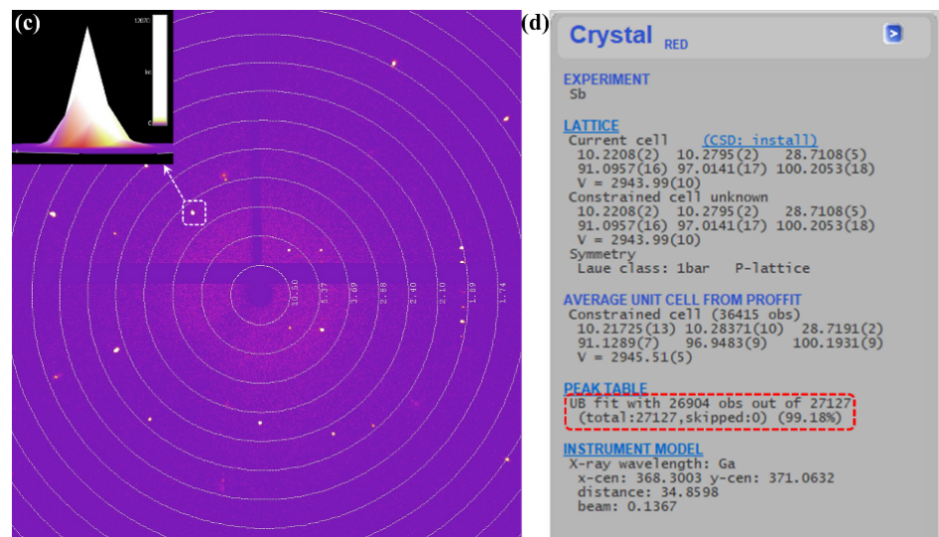


**Figure S25.** X-ray single crystal diffraction experiment after the transformation. (a-b) The diffraction frames (inset: 3D shapes of selected Bragg reflections) and the unit cell and indexation rate of compound **1** after transformation; (b-c) The diffraction frames (inset: 3D shapes of selected Bragg reflections) and the unit cell and indexation rate of compound **2** after transformation.

**Nots:** To prove the single crystal-to-single crystal transformation, we further performed X-ray single-crystal diffraction tests for the crystal after the transformation. As shown in Figure S32-33, the appearance of compound **1** and **2** after the structural transformation are clearly presented on the single-crystal diffractometer, which also still shows independent and strong diffraction points. The independent diffraction points and high indexation rate (99.89% and 99.18% for compound **1-2** after transformation, respectively) further indicate that the sample maintains good single-crystal phase after the structural transformation. Further, we conducted more detailed X-ray single-crystal diffraction test and structural refinement for the transformed sample. The unit cell parameters and good refinement results (Table. S11, *R*_1_ = 0.0572 *wR*_2_ = 0.1566, *GOF* =1.033) of **1** after transformation are almost the same as compound **2**, which accurately prove that compound **1** can undergo structural transformation to obtain single crystal of compound **2** under ethanol stimulation, which confirms the transition from **1** to **2** is single-crystal to single-crystal transformation on the atomic level. Similarly, the structure of compound **3** was obtained from the single crystal of transformed compound **2**, which proved the process of compound **2** to compound **3** is also a single-crystal to single-crystal transformation.

**(a)**

**(b)**

**Figure S26.** Comparisons of experimental PXRD patterns (a) and PL emission spectra (b) of compound **2** before and after exposure in humid air for 100 days.

****(a)

****(b)

**Figure S27.** The PXRD patterns and PL emission spectra of compound **2** after soaking various organic solvents over one day (EtOH = Ethanol, *i*-PrOH = *iso*-propanol, MeCN = Acetonitrile, PhMe = Toluene, THF = Tetrahydrofuran, EA = Ethyl acetate).

**
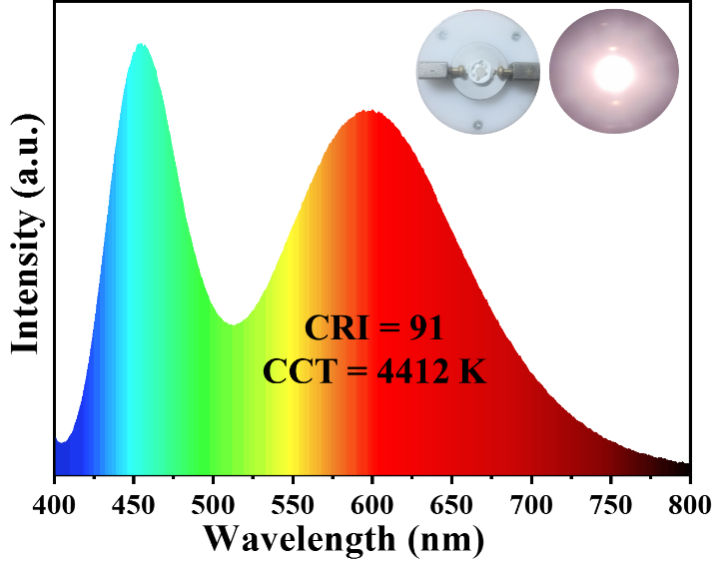
**(a)

**
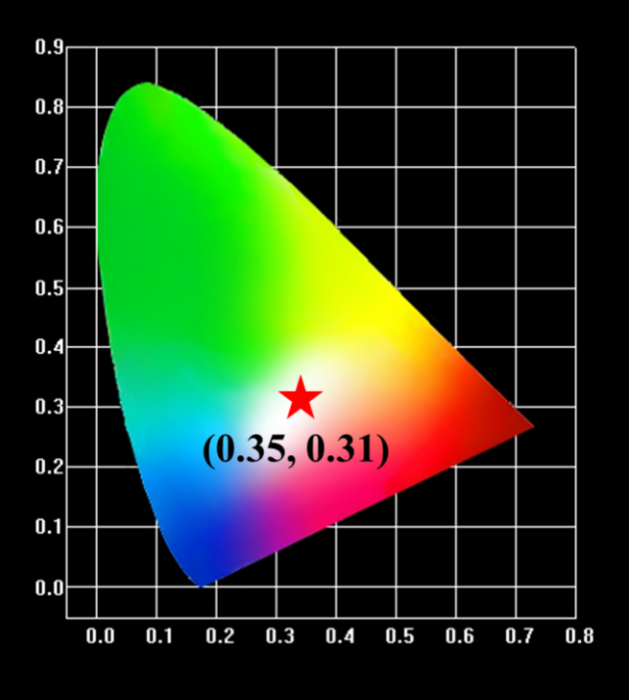
**(b)

**Figure S28.** Characterizations of white LED fabricated by mixing the yellow phosphor compound **2** and blue phosphor BaMgAl_10_O_17_:Eu^2+^ on a UV LED chip: (a) the EL spectra at 20 mA drive current with correlated color temperature of 4412 K corresponding to warm white light (inset: photograph of fabricated WLED), and the color rendering index of 91 far exceed the CRI value of commercial available WLED (< 80), (b) the CIE coordinates (0.35, 0.31) of fabricated white LED are close to the nature white light.

****(a)

****(b)

****(c)

**Figure S29.** The thermogravimetric analysis (TGA) curves of compound **1** (a), compound **2** (b) and compound **3** (c).

**Figure S30.** The experimental PXRD pattern and simulated date of compound **3** after reversible transformation between compounds **2** and **3** over 5 cycles.

**
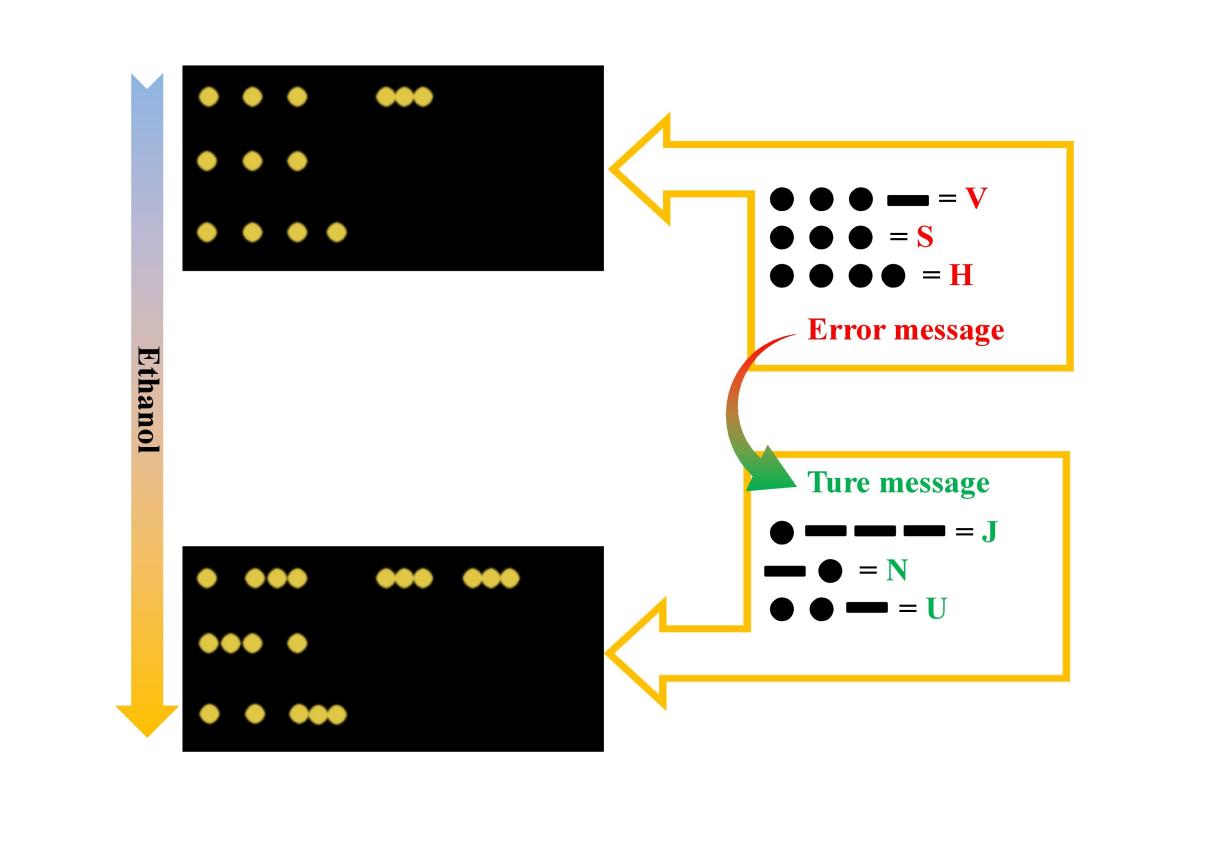
**

**Figure S31.** Illustration of single-mode digital information encryption-decryption process based on compounds **1** to **2**: the confidential digital information is encrypted in microarray dots based on combined compounds **1** and **2** according to Morse code. All the microarray dots are invisible under the sunlight to encrypt the information. Under the 365 nm UV light irradiation, only the microarray dots composed of compound **2** display yellow light emission giving error massage of “VSH”. Under the trigger of EtOH, the non-luminescent dots of **1** turn into yellow-emissive **2** with PL transformation. Therefore, only those who have the key can easily read and translate the dot-matrices into “JNU” according to Morse code.


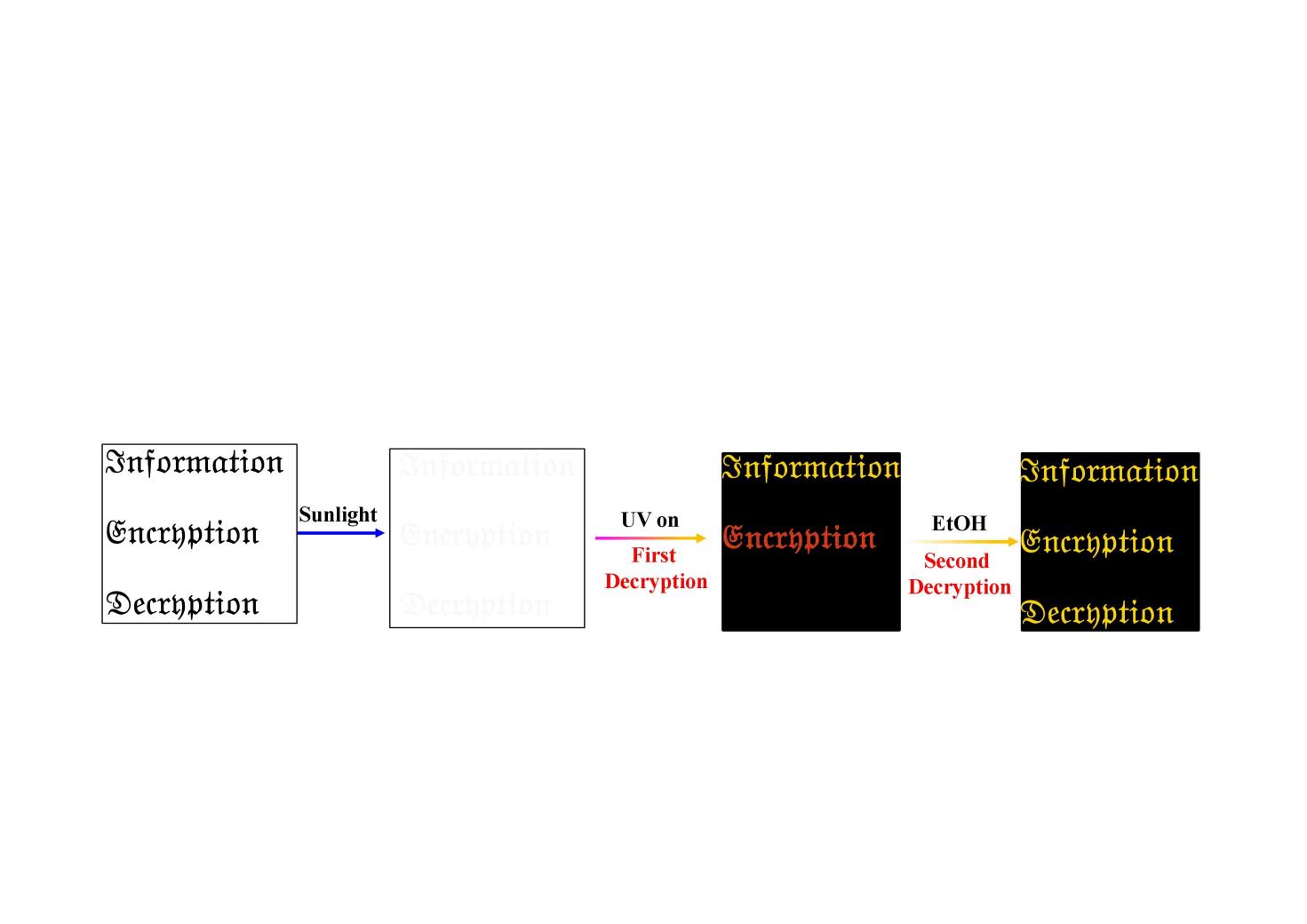


**Figure S32.** Illustration of double-mode digital information encryption-decryption process based on compounds **1** to **3**: the words of “information”, “encryption” and “decryption” are printed by using compound **2**, **3** and **1** as solid-state luminescence materials, respectively. Under the 365 nm UV light irradiation, all the printed words are invisible as information encryption. Upon the 365 nm UV light excitation, yellow color “information” and red color “encryption” appear as the first information decryption. After the trigger of EtOH, both the compounds **1** and **3** convert into compounds **2**, which enable all the printed words display yellow light emission giving final information of “information encryption decryption”. Therefore, double-mode digital information encryption-decryption process is realized.

**Figure S33.** Comparison of the PL spectra of com **2**@PTFE composite before and after heating treatment.

**Table S1**. Summary of the PL properties of single crystalline antimony perovskites at 300 K.

| **Halides** | **λ_em_ nm** | **Stocks shift nm** | **FWHM nm** | **PLQY** | **Lifetime** | **Ref.** |
| --- | --- | --- | --- | --- | --- | --- |
| **Square pyramidal [SbX_5_] based halides** | | | | | | |
| (PPN)_2_SbCl_5_ | 635 | 225 | 142 | 98.1% | 4.1 *μ*s | 7 |
| (TEBA)_2_SbCl_5_ | 590 | 250 | 140 | 98% | 13.44 *μ*s | 8 |
| (C_9_NH_20_)_2_SbCl_5_ | 590 | 210 | 119 | 98% | 4.2 *μ*s | 9 |
| **[Ph_3_EtP]_2_SbCl_5_·EtOH** | **598** | **228** | **123** | **91.5%** | **3.527 *μ*s** | **This work** |
| [C@Cs]_2_SbCl_5_ | 664 | 320 | 149 | 89% | 5.26 *μ*s | 10 |
| (Ph_4_P)_2_SbCl_5_ | 648 | 273 | 136 | 87% | 4.75 *μ*s | 11 |
| [Bmim]_2_SbCl_5_ | 583 | 215 | ∙∙∙∙ | 86.3% | 4.26 *μ*s | 12 |
| (TTA)_2_SbCl_5_ | 625 | 250 | 140 | 86% | 12.38 *μ*s | 7 |
| [C@Rb]_2_SbCl_5_ | 686 | 346 | 165 | 75% | 4.85 *μ*s | 10 |
| **[Ph_3_EtP]_2_SbCl_5_** | **642** | **277** | **146** | **64.47%** | **4.364 *μ*s** | **This work** |
| [(NH_4_)(18-crown-6)]_2_SbCl_5_ | 685 | 320 | 283 | 57% | 3.8 *μ*s | 14 |
| [C@Rb]_2_SbBr_5_ | 713 | 320 | 149 | 56% | 2.28 *μ*s | 10 |
| [Rb(18-crown-6)]_2_SbCl_5_ | 660 | 273 | 182 | 54% | 5.4 *μ*s | 13 |
| [TEMA]_2_SbCl_5_ | 636 | 280 | ∙∙∙∙ | 46% | ∙∙∙∙ | 14 |
| [Bzmim]_2_SbCl_5_ | 600 | 225 | ∙∙∙∙ | 22.3% | 2.6 *μ*s | 15 |
| [(NH_4_)(18-crown-6)]_2_SbBr_5_ | 735 | 292 | 198 | ∙∙∙∙ | 1.4 *μ*s | 13 |
| **Octahedral [SbX_6_] based halides** | | | | | | |
| [DPA]_3_SbCl_6_ | 520 | 153 | 113 | 92.7% | 2.31 *μ*s | 16 |
| [Bzmim]_3_SbCl_6_ | 525 | 183 | ∙∙∙∙ | 87.5% | 2.4 *μ*s | 15 |
| [DMPZ]_2_SbCl_6_∙Cl∙(H_2_O)_2_ | 611 | 247 | 151 | 75.9% | 2.336 *μ*s | 17 |
| [H_3_L_6_]SbBr_6_ | 530 | 170 | 110 | 55% | 2.15 *μ*s | 18 |
| [C@Ba]_4_[SbBr_6_]_2_[Sb_2_Br_8_] | 591 | 206 | 137 | 46% | 1.40 *μ*s | 10 |
| [C@Cs][C@Ba][SbBr_6_] | 643 | 264 | 173 | 45% | 1.28 *μ*s | 10 |
| (H_3_O)[TAEA]_2_[SbCl_6_]∙[Sb_2_Cl_10_]∙Cl_2_ | 517 | 165 | 110 | 45% | 22.9 *μ*s | 19 |
| [TAEA]_4_[SbCl_6_]_3_∙Cl_7_ | 580 | 200 | 140 | 43% | 17.11 *μ*s | 19 |
| [C@Rb][C@Ba][SbBr_6_] | 617 | 241 | 155 | 40% | 1.66 *μ*s | 10 |
| [TAEA]_2_(SbCl_6_)_2_∙Cl_2_ | 638 | 290 | 160 | 6% | 14.48 *μ*s | 19 |
| (PEA)_4_Bi_0.57_Sb_0.43_Br_7_∙H_2_O | 640 | 240 | 160 | 4.5% | 0.234 *μ*s | 20 |
| Rb_7_Sb_3_Cl_16_ | 560 | 195 | ∙∙∙∙ | 3.8% | 0.2 *μ*s | 21 |
| (PMA)_3_SbBr_6_ | 625 | 200 | 175 | <1% | 1.508 ns | 22 |

TTA = Tetraethylammonium; TEBA = Benzyltriethylammonium; PEA = phenethylamine, PPN = bis(triphenylphosphoranylidene)ammonium; Ph_3_EtP = triphenylethylphosphonium, DPA = dipropylamine, DMPZ = N,N′-dimethylpiperazine; Bzmim = 1-benzyl-3-methylimidazolium; Bmim = 1-Butyl-3-methylimidazolium; L = 2-(3-methyl-1Himidazol-3-ium-1-yl)acetate; C_9_NH_20_ = 1-butyl-1-methylpyrrolidinium; Ph_4_P = tetraphenylphosphonium; 4-MP = 4-methylpiperidinium; C_5_N_2_H_16_ = N-ethyl-1,3-propanediamine; PMA = C_6_H_5_CH_2_NH_3_; TEMA = methyltriethylammonium; [C@M] = 18-crown-6 metal complex cation.

**Table S2**. Summary of the applications for anti-counterfeiting, information encryption-decryption and optical logic gates of 0D antimony halide perovskites bulk crystals.

| **Halides** | **Anti-counterfeiting** | **Information encryption-decryption** | **Optical logic gates** | **SC-SC transformation** | **References** |
| --- | --- | --- | --- | --- | --- |
| **[Ph_3_EtP]_2_Sb_2_Cl_8_** | **√** | **√** | **√** | **√**  **triple conversion** | ***This work*** |
| **[Ph_3_EtP]_2_SbCl_5_·EtOH** |  |  |  |  |  |
| **[Ph_3_EtP]_2_SbCl_5_** |  |  |  |  |  |
| [Bmmim]_2_SbCl_5_ | **√** | √ | No | √  single conversion | *Angew. Chem. Int. Ed.* **2021**, 60, 23373. |
| *β*-[Bmmim]_2_SbCl_5_ |  |  |  |  |  |
| [DPA]_3_SbCl_6_ | √ | √ | No | No | *Chem. Eng. J.* **2022**, *431*, 134336. |
| BAPPSb_2_Cl_10_ | **√** | No | No | No | *Sci. Adv.* **2021**, *7*, eabg3989. |
| [Bzmim]_3_SbCl_6_ | **√** | No | No | **√**  single conversion | *Angew. Chem. Int. Ed.* **2019**, *58*, 9974. |
| [Bzmim]_2_SbCl_5_ |  |  |  |  |  |
| [TEMA]_2_SbCl_5_ | **√** | No | No | No | *Sci. Adv.* **2020**, *6*, eabc2181. |
| C_6_H_15_Cl_2_NO⋅SbCl_5_ | No | No | No | No | *Adv. Optical Mater.* **2022**, 2200591 |
| (MePPh_3_)_2_SbCl_5_ | No | No | No | No | *Angew. Chem. Int. Ed.* **2021**, 60, e202113450. |
| (DTA)_2_SbCl_5_·DTAC | No | No | No | No | *Angew. Chem. Int. Ed.* **2021**, *60*, 13548. |
| Rb_7_Sb_3_Cl_16_ | No | No | No | No | *Angew. Chem. Int. Ed.* **2020**, *59*, 14490. |
| (PEA)_4_Bi_0.57_Sb_0.43_Br_7_∙H_2_O | No | No | No | No | *Angew. Chem. Int. Ed.* **2019**, *58*, 2725. |
| (TEBA)_2_SbCl_5_ | No | No | No | No | *Chem. Mater.* **2019**, *31*, 9363. |
| (C_9_NH_20_)_2_SbCl_5_ | No | No | No | No | *Chem. Sci.* **2018**, *9*, 586. |
| (2cepyH)SbCl_4_ | No | No | No | No | *Sci. China. Chem.*, **2021**, *64*, 2111–2117. |
| (Ph_4_P)_2_SbCl_5_ | No | No | No | No | *Chem. Mater.* **2018**, *30*, 2374. |
| (TPA)_2_SbCl_5_ | No | No | No | No | *J. Mater. Chem. C*, **2021**, *9*, 12184. |
| [DMPZ]_2_SbCl_6_∙Cl∙(H_2_O)_2_ | No | No | No | No | *Adv. Opt. Mater.* **2021**, *9*, 2100556. |
| (TTA)_2_SbCl_5_ | No | No | No | No | *ACS Mater. Lett.* **2020**, *2*, 633. |
| (PPN)_2_SbCl_5_ | No | No | No | No |  |
| (PMA)_3_SbBr_6_ | No | No | No | No | *J. Mater. Chem. C* **2018**, *6*, 2801. |
| (TMA)_2_SbCl_5_·DMF | No | No | No | No | *J. Phys. Chem. Lett.* **2021**, *12*, 7091. |
| [TAEA]_4_[SbCl_6_]_3_∙Cl_7_ | No | No | No | No | *J. Mater. Chem. C* **2021**, *9*, 348. |
| [TAEA]_2_(SbCl_6_)_2_∙Cl_2_ | No | No | No | No |  |
| (H_3_O)[TAEA]_2_[SbCl_6_]∙[Sb_2_Cl_10_]∙Cl_2_ | No | No | No | No |  |
| [Rb(18-crown-6)]_2_SbCl_5_ | No | No | No | No | *J. Phys. Chem. C* **2020**, *124*, 11625. |
| [C@Cs]_2_SbCl_5_ | No | No | No | No | *ACS Mater. Lett.* **2020**, *2*, 845. |
| [(NH_4_)(18-crown-6)]_2_SbCl_5_ | No | No | No | No |  |
| [C@Rb]_2_SbCl_5_ | No | No | No | No |  |
| [C@Rb]_2_SbBr_5_ | No | No | No | No |  |
| [C@Ba]_4_[SbBr_6_]_2_[Sb_2_Br_8_] | No | No | No | No |  |
| [C@Cs][C@Ba][SbBr_6_] | No | No | No | No |  |
| [C@Rb][C@Ba][SbBr_6_] | No | No | No | No |  |
| (TMEDA)SbI_5_ | No | No | No | No | *Adv. Opt. Mater.* **2021**, 2101333. |
| [H_3_L_6_]SbBr_6_ | No | No | No | No | *J. Mater. Chem. C* **2020**, *8*, 7300. |
| H_3_SbCl_6_(L)_6_ | No | No | No | No | *Chem. Commun*. **2021**, *57*, 1754. |
| [Emim]_8_[SbCl_6_]_2_[SbCl_5_] | No | No | No | No | *Dalton Trans.* **2021**, *50*, 3586. |
| [Bmim]_2_SbCl_5_ | No | No | No | No | *Chem. Commun.* **2015**, *51*, 3094. |
| [(NH_4_)(18-crown-6)]_2_SbBr_5_ | No | No | No | No | *J. Phys. Chem. C* **2020**, *124*, 11625. |
| (C_3_H_12_N_2_)_2_Sb_2_Cl_10_ | No | No | No | No | *Inorg. Chem.* **2021**, *60*, 11429. |

**Table S3**. Crystal Data and Structural Refinements for [Ph_3_EtP]_2_Sb_2_Cl_8_ (**1**), [Ph_3_EtP]_2_SbCl_5_·EtOH (**2**) and [Ph_3_EtP]_2_SbCl_5_ (**3**).

| compound | [Ph_3_EtP]_2_Sb_2_Cl_8_ (**1**) | [Ph_3_EtP]_2_SbCl_5_·EtOH (**2**) | [Ph_3_EtP]_2_SbCl_5_ (**3**) |
| --- | --- | --- | --- |
| chemical formula | C_40_H_40_Cl_8_P_2_Sb_2_ | C_42_H_45_Cl_5_OP_2_Sb | C_40_H_40_Cl_5_P_2_Sb |
| fw | 1109.76 | 926.72 | 881.66 |
| Temp (K) | 100.03(10) | 293(2) | 100.00(10) |
| Crystal system | monoclinic | monoclinic | triclinic |
| Space group | *P*2_1_/n | *C*2/*c* | *P*-1 |
| *a* (Å) | 10.6806(5) | 22.0454(7) | 10.2134(6) |
| *b* (Å) | 11.1509 (6) | 16.0378(6) | 10.2800(6) |
| *c* (Å) | 37.9082 (2) | 13.3107(5) | 28.7265(13) |
| *α* (°) | 90 | 90 | 91.1245(4) |
| *β* (°) | 92.7710(4) | 120.080(10) | 96.9622(4) |
| *γ* (°) | 90 | 90 | 100.1675(5) |
| *V*(Å^3^) | 4509.52(3) | 4072.3(3) | 2944.25(3) |
| Z | 4 | 4 | 3 |
| *D*_calcd_ (g∙cm^-3^) | 1.635 | 1.512 | 1.492 |
| *μ* (mm^-1^) | 14.751 | 1.117 | 6.435 |
| *F* (000) | 2192.0 | 1884.0 | 1338.0 |
| Reflections collected | 110558 | 43733 | 41042 |
| Unique reflections | 9344 | 6227 | 13280 |
| GOF on *F* ^2^ | 1.060 | 1.066 | 1.064 |
| ^a^*R*_1_, *wR*_2_ (*I* > 2*σ*(*I*)) | 0.0211/0.0516 | 0.0171/0.0420 | 0.0434/0.0988 |
| *^b^R*_1_, *wR*_2_ (all data) | 0.0220/0.0522 | 0.0193/0.0429 | 0.0463/0.1001 |

*^a^R_1_* = ∑||*F*_o_| - |*F*_c_||/∑|*F*_o_|. *^b^wR*_2_ = [∑*w*(*F*_o_^2^ - *F*_c_^2^)^2^/∑*w*(*F*_o_^2^)^2^]^1/2^.

**Table S4**. Selected bond lengths (Å) and bond angles (°) for compound **1**.

| **Compound 1** | | | |
| --- | --- | --- | --- |
| Sb1-Cl1^1^ | 3.1646(6) | Sb2-Cl3 | 3.4795(5) |
| Sb1-Cl1 | 2.5838(5) | Sb2-Cl4 | 2.9693(5) |
| Sb1-Cl2 | 2.3818(5) | Sb2-Cl5 | 2.6606(5) |
| Sb1-Cl3 | 2.4350(5) | Sb2-Cl6 | 2.5516(5) |
| Sb1-Cl4 | 2.6655(5) | Sb2-Cl7 | 2.4422(5) |
| Sb1-Cl5 | 3.1364(5) | Sb2-Cl8 | 2.3852(4) |
| Cl1-Sb1-Cl1^1^ | 88.202(17) | Cl4-Sb2-Cl3 | 67.102(12) |
| Cl1-Sb1-Cl4 | 174.25(2) | Cl5-Sb2-Cl3 | 71.018(13) |
| Cl1-Sb1-Cl5 | 106.69(2) | Cl5-Sb2-Cl4 | 80.644(14) |
| Cl2-Sb1-Cl1^1^ | 82.77(2) | Cl6-Sb2-Cl3 | 110.556(14) |
| Cl2-Sb1-Cl1 | 87.40(3) | Cl6-Sb2-Cl4 | 96.277(16) |
| Cl2-Sb1-Cl3 | 90.403(19) | Cl6-Sb2-Cl5 | 175.762(17) |
| Cl2-Sb1-Cl4 | 87.78(2) | Cl7-Sb2-Cl3 | 110.224(15) |
| Cl2-Sb1-Cl5 | 162.54(2) | Cl7-Sb2-Cl4 | 171.519(18) |
| Cl3-Sb1-Cl1 | 87.555(18) | Cl7-Sb2-Cl5 | 90.875(18) |
| Cl3-Sb1-Cl1^1^ | 172.118(19) | Cl7-Sb2-Cl6 | 92.19(2) |
| Cl3-Sb1-Cl4 | 89.336(16) | Cl8-Sb2-Cl3 | 149.189(14) |
| Cl3-Sb1-Cl5 | 80.186(15) | Cl8-Sb2-Cl4 | 87.445(15) |
| Cl4-Sb1-Cl1^1^ | 94.310(15) | Cl8-Sb2-Cl5 | 88.548(16) |
| Cl4-Sb1-Cl5 | 77.513(13) | Cl8-Sb2-Cl6 | 88.405(16) |
| Cl5-Sb1-Cl1^1^ | 107.415(19) | Cl8-Sb2-Cl7 | 92.370(17) |
| ^1^1-X,2-Y,1-Z | | | |

**Table S5**. Comparison of the bond length and angles in [SbCl_5_]^2-^ square pyramid at ground and excited states of [Ph_3_EtP]_2_SbCl_5_·EtOH (**2**).

| **Bond length (Å)** | **Ground** | **Excited** |
| --- | --- | --- |
| Sb1-Cl1 | 2.3688(4) | 2.490 |
| Sb1-Cl2^1^ | 2.6209(3) | 2.963 |
| Sb1-Cl2 | 2.6209(3) | 2.963 |
| Sb1-Cl3^1^ | 2.6197(3) | 2.863 |
| Sb1-Cl3 | 2.6197(3) | 2.863 |
| **Bond angle (°)** | **Ground** | **Excited** |
| Cl1-Sb1-Cl2^1^ | 89.493(6) | 73.578 |
| Cl1-Sb1-Cl2 | 89.492(6) | 73.578 |
| Cl1-Sb1-Cl3^1^ | 86.630(6) | 71.520 |
| Cl1-Sb1-Cl3 | 86.631(6) | 71.520 |
| Cl3^1^-Sb1-Cl2 | 88.929(9) | 88.674 |
| Cl3-Sb1-Cl2^1^ | 88.929(9) | 88.674 |
| Cl3-Sb1-Cl2 | 91.010(9) | 81.020 |
| Cl3^1^-Sb1-Cl2^1^ | 91.013(9) | 81.020 |
| ^1^1-X, +Y, 3/2-Z | | |

**Table S6**. Comparison of the bond length and angles in [SbCl_5_]^2-^ square pyramid at ground and excited states of [Ph_3_EtP]_2_SbCl_5_ (**3**).

| **Bond length (Å)** | **Ground** | **Excited** |
| --- | --- | --- |
| Sb1-Cl4 | 2.378 | 2.380 |
| Sb1-Cl5 | 2.691 | 2.690 |
| Sb1-Cl2 | 2.553 | 2.553 |
| Sb1-Cl1 | 2.609 | 2.613 |
| Sb1-Cl3 | 2.575 | 2.578 |
| Sb2-Cl8 | 2.604 | 2.572 |
| Sb2-Cl8^1^ | 2.604 | 2.572 |
| Sb2-Cl7 | 2.592 | 2.601 |
| Sb2-Cl6 | 2.240 | 2.350 |
| Sb2-Cl6^1^ | 2.240 | 2.350 |
| **Bond angle (°)** | **Ground** | **Excited** |
| Cl4-Sb1-Cl5 | 85.26 | 85.23 |
| Cl4-Sb1-Cl2 | 88.39 | 88.42 |
| Cl4-Sb1-Cl1 | 87.20 | 87.25 |
| Cl4-Sb1-Cl3 | 86.17 | 86.12 |
| Cl2-Sb1-Cl1 | 93.44 | 93.44 |
| Cl2-Sb1-Cl3 | 90.03 | 90.05 |
| Cl1-Sb1-Cl5 | 86.91 | 86.92 |
| Cl3-Sb1-Cl5 | 88.90 | 88.88 |
| Cl7-Sb2-Cl8 | 91.07 | 95.76 |
| Cl7-Sb2-Cl8^1^ | 88.93 | 95.36 |
| Cl6-Sb2-Cl8^1^ | 93.90 | 96.58 |
| Cl6^1^-Sb2-Cl8 | 93.90 | 96.58 |
| Cl6^1^-Sb2-Cl8^1^ | 86.10 | 88.24 |
| Cl6-Sb2-Cl8 | 86.10 | 88.24 |
| Cl6^1^-Sb2-Cl7 | 89.81 | 85.38 |
| Cl6-Sb2-Cl7 | 90.19 | 100.53 |
| ^1^ -X, -1-Y, 2-Z | | |

**Table S7.** Comparison of the distortion degrees of [SbCl_5_]^2-^ square pyramid at ground and excited states of [Ph_3_EtP]_2_SbCl_5_·EtOH (**2**) and [Ph_3_EtP]_2_SbCl_5_ (**3**).

|  | | **Ground state** | | **Excited state** | |
| --- | --- | --- | --- | --- | --- |
|  |  | **Δ*d*** | ***σ^2^*** | **Δ*d*** | ***σ^2^*** |
| [Ph_3_EtP]_2_SbCl_5_·EtOH | Sb1 | 1.53 × 10^-3^ | 3.93521 | 3.52 × 10^-3^ | 198.169 |
| [Ph_3_EtP]_2_SbCl_5_ | Sb1 | 1.62 × 10^-3^ | 10.0308 | 1.60 × 10^-3^ | 10.0647 |
|  | Sb2 | 3.21 × 10^-3^ | 9.02473 | 2.10 × 10^-3^ | 40.9886 |

**Table S8.** Hydrogen bonds data for compound **1.**

| **D-H···A** | **d(D-H)** | **d(H···A)** | **d(D···A)** | **<(DHA)** |
| --- | --- | --- | --- | --- |
| C6-H6∙∙∙Cl2 | 0.93 | 2.74 | 3.670(2) | 174 |
| C17-H17∙∙∙Cl5 | 0.93 | 2.73 | 3.515(2) | 143 |
| C18-H18∙∙∙Cl4 | 0.93 | 2.77 | 3.619(2) | 152 |
| C37-H37∙∙∙Cl1 | 0.93 | 2.79 | 3.546(2) | 139 |
| C40-H40∙∙∙Cl6 | 0.93 | 2.71 | 3.492(2) | 142 |

**Table S9.** Hydrogen bonds data for compound **2.**

| **D-H···A** | **d(D-H)** | **d(H···A)** | **d(D···A)** | **<(DHA)** |
| --- | --- | --- | --- | --- |
| O1-H1∙∙∙Cl2 | 0.82 | 2.58 | 3.340(2) | 155 |
| C2-H2A∙∙∙Cl3 | 0.97 | 2.80 | 3.619(1) | 142 |
| C4-H4∙∙∙Cl3 | 0.93 | 2.76 | 3.654(1) | 161 |
| C13-H13∙∙∙Cl3 | 0.93 | 2.81 | 3.511(2) | 133 |

**Table S10.** Hydrogen bonds data for compound **3.**

| **D-H···A** | **d(D-H)** | **d(H···A)** | **d(D···A)** | **<(DHA)** |
| --- | --- | --- | --- | --- |
| C5-H5∙∙∙Cl5 | 0.93 | 2.74 | 3.483(3) | 137 |
| C15-H15∙∙∙Cl3 | 0.93 | 2.77 | 3.466(2) | 133 |
| C19-H19A∙∙∙Cl5 | 0.97 | 2.50 | 3.428(2) | 159 |
| C22-H22∙∙∙Cl8 | 0.93 | 2.80 | 3.547(3) | 138 |
| C25-H25∙∙∙Cl7 | 0.93 | 2.81 | 3.614(3) | 146 |
| C30-H30∙∙∙Cl7 | 0.93 | 2.77 | 3.539(3) | 141 |
| C34-H34∙∙∙Cl1 | 0.93 | 2.71 | 3.548(3) | 151 |
| C36-H36∙∙∙Cl4 | 0.93 | 2.82 | 3.387(2) | 120 |
| C39-H39A∙∙∙Cl8 | 0.97 | 2.81 | 3.538(2) | 132 |
| C43-H43∙∙∙Cl6 | 0.93 | 2.74 | 3.586(3) | 151 |
| C45-H45∙∙∙Cl8 | 0.93 | 2.74 | 3.652(2) | 167 |
| C51-H51∙∙∙Cl3 | 0.93 | 2.66 | 3.528(2) | 156 |
| C59-H59A∙∙∙Cl8 | 0.97 | 2.75 | 3.675(2) | 159 |
| C59-H59B∙∙∙Cl3 | 0.97 | 2.69 | 3.624(2) | 162 |

**Table. S11.** Crystal data and structure refinement of compound **1** after transformation.

| **Crystal data and structure refinement of 1 after transformation** | |
| --- | --- |
| Space group | *C*2*/c* |
| *a* (Å) | 22.0370(3) |
| *b* (Å) | 16.0243(1) |
| *c* (Å) | 13.3143(2) |
| *α* (°) | 90 |
| *β* (°) | 120.062(2) |
| *γ* (°) | 90 |
| *V* (Å^3^) | 4069.20(11) |
| Reflections collected | 24315 |
| GOF on *F* ^2^ | 1.033 |
| *^a^R*_1_, *wR*_2_ (*I* >2*σ*(*I*)) | 0.0572/0.1566 |
| *^b^R*_1_, *wR*_2_ (all data) | 0.0578/0.1576 |

**Table S12.** Calculated energy for compounds **1**-**3**, EtOH and SbCl_3_.

| **Compound** | **Energy / Hatree** |
| --- | --- |
| [Ph_3_EtP]_2_Sb_2_Cl_8_ | -5923.35 |
| [Ph_3_EtP]_2_SbCl_5_·EtOH | -4767.58 |
| [Ph_3_EtP]_2_SbCl_5_ | -4209.57 |
| EtOH | -155.03 |
| SbCl_3_ | -1386.10 |

**References**

1. G. M. Sheldrick, *Acta Crystallogr. A* **2015**, *71*, 3-8.
2. M. D. Segall, P. J. D. Lindan, M. J. Probert, C. J. Pickard, P. J. Hasnip, S. J. Clark, M. C. Payne, *J. Phys.: Condens. Matter* **2002**, *14*, 2717-2744.
3. G. Kresse, J. Furthmüller, *Phys. Rev. B* **1996**, *54*, 11169-11186.
4. J. P. Perdew, K. Burke, M. Ernzerhof, *Phys. Rev. Lett.* **1996**, *77*, 3865-3868.
5. G. Kresse, D. Joubert, *Phys. Rev. B* **1999**, *59*, 1758-1775.
6. Gaussian 16, Revision A.03, M. J. Frisch, G. W. Trucks, H. B. Schlegel, G. E. Scuseria, M. A. Robb, *et. al*, Wallingford CT, 2016.
7. Q. He, C. Zhou, L. Xu, S. Lee, X. Lin, J. Neu, M. Worku, M. Chaaban, B. Ma, *ACS Mater. Lett.* **2020**, *2*, 633-638.
8. Z. Li, Y. Li, P. Liang, T. Zhou, L. Wang, R.-J. Xie, *Chem. Mater.* **2019**, *31*, 9363-9371.
9. C. Zhou, H. Lin, Y. Tian, Z. Yuan, R. Clark, B. Chen, L. J. van de Burgt, J. C. Wang, Y. Zhou, K. Hanson, Q. J. Meisner, J. Neu, T. Besara, T. Siegrist, E. Lambers, P. Djurovich, B. Ma, *Chem. Sci.* **2018**, *9*, 586-593.
10. V. Morad, S. Yakunin, M. V. Kovalenko, *ACS Mater. Lett.* **2020**, *2*, 845-852.
11. C. Zhou, M. Worku, J. Neu, H. Lin, Y. Tian, S. Lee, Y. Zhou, D. Han, S. Chen, A. Hao, P. I. Djurovich, T. Siegrist, M.-H. Du, B. Ma, *Chem. Mater.* **2018**, *30*, 2374-2378.
12. Z.-P. Wang, J.-Y. Wang, J.-R. Li, M.-L. Feng, G.-D. Zou, X.-Y. Huang, *Chem. Commun.* **2015**, *51*, 3094-3097.
13. J. Xu, S. Li, C. Qin, Z. Feng, Y. Du, *J. Phys. Chem. C* **2020**, *124*, 11625-11630.
14. Z. Wang, D. Xie, F. Zhang, J. Yu, X. Chen, C. P. Wong, *Sci. Adv.* **2020**, *6*, eabc2181.
15. Z. Wang, Z. Zhang, L. Tao, N. Shen, B. Hu, L. Gong, J. Li, X. Chen, X.-Y. Huang, *Angew. Chem. Int. Ed.* **2019**, *58*, 9974-9978.
16. J.-Q. Zhao, H.-S. Shi, L.-R. Zeng, H. Ge, Y.-H. Hou, X.-M. Wu, C.-Y. Yue, X.-W. Lei, *Chem. Eng. J.* **2022**, *431*, 134336.
17. J.-Q. Zhao, M.-F. Han, X.-J. Zhao, Y.-Y. Ma, C.-Q. Jing, H.-M. Pan, D.-Y. Li, C.-Y. Yue, X.-W. Lei, *Adv. Opt. Mater.* **2021**, *19*, 2100556.
18. F. Lin, H. Wang, W. Liu, J. Li, *J. Mater. Chem. C* **2020**, *8*, 7300-7303.
19. A. Biswas, R. Bakthavatsalam, B. P. Mali, V. Bahadur, C. Biswas, S. S. K. Raavi, R. G. Gonnade, J. Kundu, *J. Mater. Chem. C* **2021**, *9*, 348-358.
20. R. Zhang, X. Mao, Y. Yang, S. Yang, W. Zhao, T. Wumaier, D. Wei, W. Deng, K. Han, *Angew. Chem. Int. Ed.* **2019**, *58*, 2725-2729.
21. B. M. Benin, K. M. McCall, M. Wörle, V. Morad, M. Aebli, S. Yakunin, Y. Shynkarenko, M. V. Kovalenko, *Angew. Chem. Int. Ed.* **2020**, *59*, 14490-14497.
22. A. Khan, A. Zeb, L. Li, W. Zhang, Z. Sun, Y. Wang, J. Luo, *J. Mater. Chem. C* **2018**, *6*, 2801-2805.
